# Analysis of the efficacy of SGLT2 inhibitors using semi-mechanistic model

**DOI:** 10.3389/fphar.2014.00218

**Published:** 2014-10-13

**Authors:** Oleg Demin, Tatiana Yakovleva, Dmitry Kolobkov, Oleg Demin

**Affiliations:** ^1^Laboratory Alpha, Institute for Systems Biology MoscowMoscow, Russia; ^2^Institute for Systems Biology MoscowMoscow, Russia

**Keywords:** SGLT-2, systems pharmacology modeling, Type 2 diabetes mellitus (T2DM), dapagliflozin

## Abstract

The Renal sodium-dependent glucose co-transporter 2 (SGLT2) is one of the most promising targets for the treatment of type 2 diabetes. Two SGLT2 inhibitors, dapagliflozin, and canagliflozin, have already been approved for use in USA and Europe; several additional compounds are also being developed for this purpose. Based on the *in vitro* IC_50_ values and plasma concentration of dapagliflozin measured in clinical trials, the marketed dosage of the drug was expected to almost completely inhibit SGLT2 function and reduce glucose reabsorption by 90%. However, the administration of dapagliflozin resulted in only 30–50% inhibition of reabsorption. This study was aimed at investigating the mechanism underlying the discrepancy between the expected and observed levels of glucose reabsorption. To this end, systems pharmacology models were developed to analyze the time profile of dapagliflozin, canagliflozin, ipragliflozin, empagliflozin, and tofogliflozin in the plasma and urine; their filtration and active secretion from the blood to the renal proximal tubules; reverse reabsorption; urinary excretion; and their inhibitory effect on SGLT2. The model shows that concentration levels of tofogliflozin, ipragliflozin, and empagliflozin are higher than levels of other inhibitors following administration of marketed SGLT2 inhibitors at labeled doses and non-marketed SGLT2 inhibitors at maximal doses (approved for phase 2/3 studies). All the compounds exhibited almost 100% inhibition of SGLT2. Based on the results of our model, two explanations for the observed low efficacy of SGLT2 inhibitors were supported: (1) the site of action of SGLT2 inhibitors is not in the lumen of the kidney's proximal tubules, but elsewhere (e.g., the kidneys proximal tubule cells); and (2) there are other transporters that could facilitate glucose reabsorption under the conditions of SGLT2 inhibition (e.g., other transporters of SGLT family).

## Introduction

Type 2 diabetes mellitus (T2DM) is a metabolic disorder that is characterized by hyperglycemia resulting from insulin resistance and relative lack of insulin. Current therapies for T2DM primarily address endocrine pathogenesis of insulin resistance and b-cell dysfunction. Consequently, many patients receive multiple glucose-lowering therapies and eventually require exogenous insulin administration. However, in clinical practice patients often fail to meet the targets for glycemic control (Ali et al., 2013), most frequently because of adverse effects–including hypoglycemia and weight gain–caused by therapeutic agents (Neumiller, 2014). Therefore, in order to avoid direct influence on insulin resistance and b-cell dysfunction, T2DM can be treated with inhibition of glucose reabsorption.

Glucose is reabsorbed to blood in the proximal tubules of the kidneys during the formation of primary urine. Where the reabsorption process is inhibited, glucose is excreted in urine and blood glucose concentration is reduced. Patients treated with inhibition of glucose reabsorption have a low risk of hypoglycemia because the mechanism of the treatment is independent of insulin release or endogenous glucose production (Misra, 2013). In the kidneys, families of glucose transporters and sodium-dependent glucose co-transporters are involved in glucose reabsorption. For example, sodium-dependent glucose co-transporter 2 (SGLT2) is thought to contribute to 90% of glucose reabsorption in kidneys (Liu et al., 2012), which makes it a promising target in T2DM therapy.

Currently, two SGLT2 inhibitors are marketed (dapagliflozin, canagliflozin) and several more are in development (e.g., empagliflozin, tofogliflozin). Based on inhibitory concentration 50% (IC50) values measured *in vitro* (Ohtake et al., 2012) and plasma concentrations measured in clinical trials (Yang et al., 2013), the marketed dosage of dapagliflozin was predicted to inhibit SGLT2 almost completely and thereby reduce glucose reabsorption by 90%. However, clinical trials demonstrated that dapagliflozin induced 50–80 g of urinary glucose excretion per day, which corresponded to only 30–50% inhibition of reabsorption (Komoroski et al., 2009a; Kasichayanula et al., 2011a). To explain these findings, several hypotheses were published (Liu et al., 2012). The following hypotheses explain the lower than predicted efficacy of SGLT2 inhibitors:

1. The concentration of the compound in the lumen of the kidney's proximal tubules (the potential site of inhibition) is low.
2. The site of action of the SGLT2 inhibitors is in the proximal tubule cells of the kidney, but not in the lumen.
3. Other transporters facilitate glucose reabsorption under conditions of SGLT2 inhibition.

To explore these hypotheses further, it is possible to adopt a systems pharmacology modeling (SPM) approach.

Many mathematical models that describe development and treatment of T2DM appear in the literature, and Ajmera et al. (2013) present a detailed review of these models. Several mathematical models describing SGLT2 inhibitors are also presented. For example, three models describe the pharmacokinetics (PK) and pharmacodynamics (PD) of SGLT2 inhibitors in animals (Yamaguchi et al., 2011, 2012, 2013), two population PK models exist for empagliflozin (Riggs et al., 2013) and dapagliflozin (van der Walt et al., 2013), and Maurer et al. (2011) describes a PK/PD model for dapagliflozin in rats and humans. However, a model that describes the concentration of SGLT2 inhibitors at the potential site of action (i.e., the lumen of proximal tubule in the kidneys) is yet to be published. The level of a compound in plasma may differ significantly from that in the kidneys; therefore, prediction of the concentration of SGLT2 inhibitors in the lumen of the kidney's proximal tubules is important for understanding the PD effect of the drug.

The aim of this study was to construct a model that describes the active secretion of SGLT2 inhibitors from plasma into the lumen of the proximal tubules, reverse reabsorption, and urinary excretion. Using an SPM approach, our objective was to test the hypotheses used to explain the discrepancy between expected and observed levels of glucose reabsorption following administrations of SGLT2 inhibitors (see above). We also aimed to compare the efficacies of different SGLT2 inhibitors by simulating their concentration level in the lumen of the kidney's proximal tubules and estimating the level of inhibition produced during treatment in humans.

## Methods

**Figure 1:**
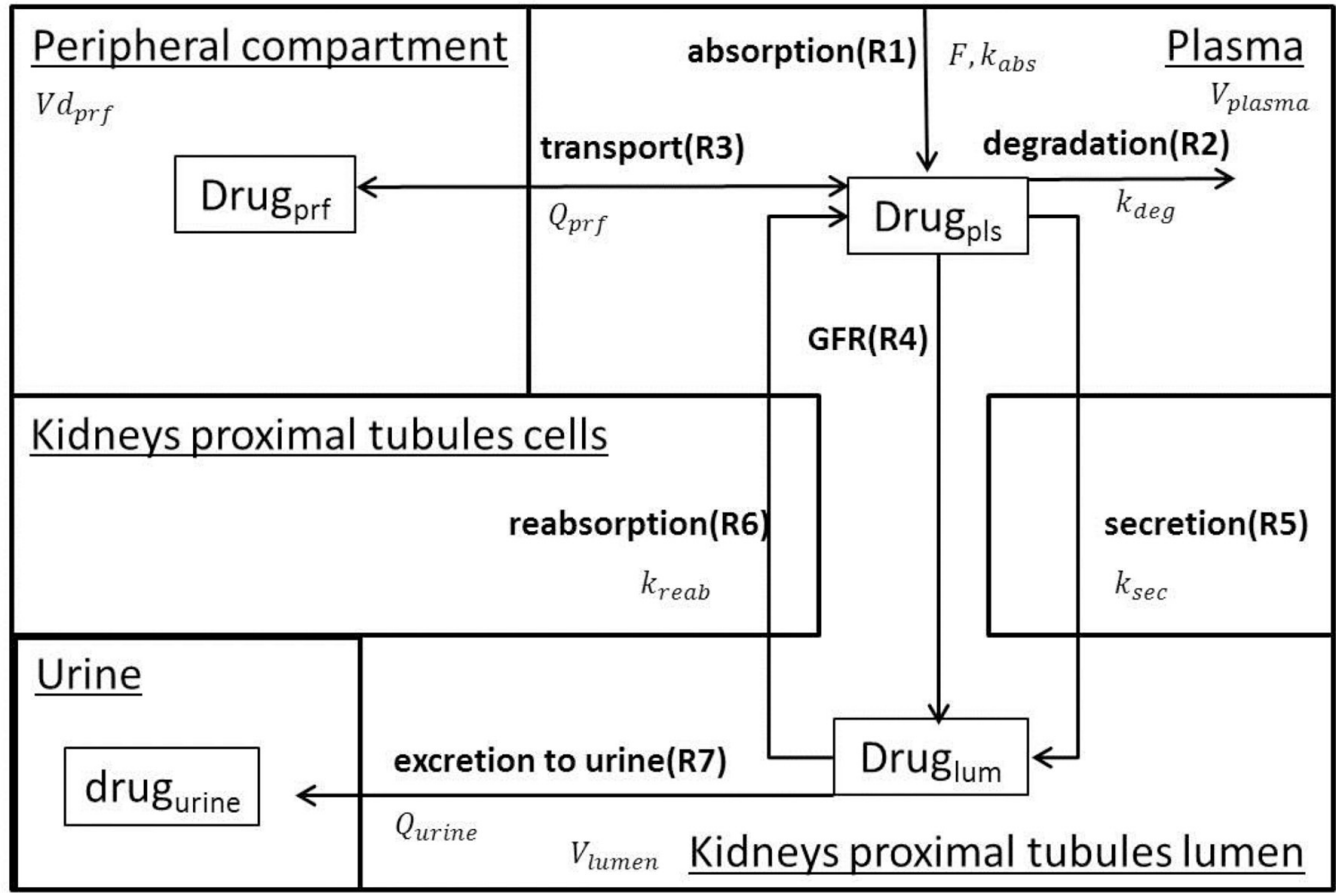
**Model Scheme**. The model describes oral drug administration, degradation, transport between plasma and peripheral compartment (for dapagliflozin, ipragliflozin, and tofogliflozin), glomerular filtration, secretion from plasma to the lumen of the kidney's proximal tubules, reverse reabsorption, and urinary excretion.

A family of semi-mechanistic PK/PD models was developed to describe administration, degradation, transport, glomerular filtration, active secretion, reverse reabsorption, and urinary excretion of 5 SGLT2 inhibitors (dapagliflozin, canagliflozin, ipragliflozin, empagliflozin, tofogliflozin). The assumptions used for model development are presented in Table 1. The models describe the PK of the drugs, inhibition of glucose reabsorption mediated by SGLT2, and levels of inhibition in transporters of the SGLT family. The models for dapagliflozin, ipragliflozin, and tofogliflozin, which have identical structures, include 4 compartments: plasma, peripheral compartment (tissues, organs), lumen of the kidney's proximal tubules, and urine. The models for canagliflozin and empagliflozin have the same structure and include 3 compartments: plasma, lumen of the kidney's proximal tubules, and urine. The rate equations for each model are similar, but many of the parameter values are specific to a particular drug. We chose to add the peripheral compartment, and a rate equation describing transport between plasma and peripheral compartment, to models describing activity of dapagliflozin, ipragliflozin, and tofogliflozin in order to achieve a better description of plasma PK data (see “Models verification strategy” Section in Supplementary Materials). The effects of compounds on SGLT2 and other transporters are described as functions of inhibitor concentration in the lumen of the kidney's proximal tubules. Schemes of the models are presented in Figure 1. Using the family of models, we were able to quantify, analyze, and compare PK and PD characteristics of the drugs. A system of ordinary differential equations, rate equations, and explicit functions are presented in Table 2. Model development and fitting procedures were performed using the DBSolve Optimum package (Gizzatkulov et al., 2010).

**Table 1:** **Assumptions for models development**.

**#**	**Assumption**
1	There is active secretion of compounds from blood to the lumen of the kidney's proximal tubules and reverse reabsorption (Liu et al., 2012)
2	Only an unbound drug could be filtrated or secreted from plasma to the lumen of the kidneys
3	The urinary excretion (Q_urine_) is similar for all compounds
4	Delay (lag) in absorption (Nerella et al., 1993; van der Walt et al., 2013) is included in all models to create a more precise description of plasma PK data
5	The site of action for SGLT2 inhibitors is in the lumen of the kidney's proximal tubules
6	The maximal inhibition level of SGLT2 (Imax) induced by compounds is equal to 1 (100%) (Grempler et al., 2012)
7	All rate equations are to be described as mass action law

**Table 2:** **Differential equations, rate equations and explicit functions**.

**#**	**Equation**	**Description**
**DIFFERENTIAL EQUATIONS**		
D1	$\frac{d(drug_{int})}{dt}=-R_{abs}$	Drug amount (mg) in intestine (*drug_int_*).
		There is a fixed delay in absorption (*lag_abs_*) implemented in the model explicitly in accordance with method proposed by (Nerella et al., 1993). Initial conditions:
		$\begin{array}{l} drug_{int}\left(0<t<lag_{abs}\right)=0,\\ drug_{int}\left(t=lag_{abs}\right)=F*Dose \end{array}$
		where *F* and *Dose* are bioavailability of a drug and dose administered
D2	$\begin{array}{l} \frac{d\left(Drug_{pls}\right)}{dt}=(R_{abs}-R_{deg}-R_{pls\_to\_\;prf}-R_{gfr}\\ \;-R_{secretion}+R_{reabsorption})/V_{plasma} \end{array}$	Drug concentration (mg/L) in plasma (*Drug_pls_*). Initial conditions:
		$Drug_{pls}\left(0\right)=0$
D3	$\frac{d\left(Drug_{prf}\right)}{dt}=(R_{pls\_to\_prf})/Vd_{prf}$	Drug concentration (mg/L) in peripheral compartment (*Drug_prf_*). The variable and rate equation *V_pls_to_prf_* are included in dapagliflozin, ipragliflozin and tofogliflozin models only. Initial conditions:
		$Drug_{prf}\left(0\right)=0$
D4	$\begin{array}{l} \frac{d\left(Drug_{lum}\right)}{dt}=(R_{gfr}+R_{secretion}\\ \;-R_{reabsorption}-R_{urine})/V_{lumen} \end{array}$	Drug concentration (mg/L) in kidneys proximal tubules lumen (*Drug_lum_*). Initial conditions:
		$Drug_{lum}\left(0\right)=0$
D5	$\frac{d\left(drug_{urine}\right)}{dt}=R_{urine}$	Drug amount (mg) in urine (*drug_urine_*). Value of the variable is set equal to zero each 24 h to calculate amount of compound recovered in urine during 24 h. Initial conditions:
		$drug_{urine}\left(0\right)=0$
**RATE EQUATIONS**		
R1	$R_{abs}=k_{abs}*drug_{int}$	Drug absorption from gastrointestinal tract
R2	$R_{deg}=V_{plasma}*k_{deg}*fup*Drug_{pls}$	Drug degradation / metabolism in plasma
R3	$R_{pls\_to\_prf}=Q_{prf}*(fup*Drug_{pls}-Drug_{prf})$	Drug transport between plasma and peripheral compartment
R4	$R_{gfr}=GFR*fup*Drug_{pls}$	Drug glomerular filtration
R5	$R_{secretion}=V_{plasma}*k_{sec}*fup*Drug_{pls}$	Drug secretion from plasma to kidneys proximal tubules lumen
R6	$R_{reabsorption}=V_{lumen}*k_{reab}*Drug_{lum}$	Drug reabsorption from kidneys proximal tubules lumen into plasma
R7	$R_{urine}=Q_{urine}*Drug_{lum}$	Drug urinary excretion
**EXPLICIT FUNCTIONS**		
E1	$Drug_{plasma\_total}^{ng/ml}=Drug_{pls}*10^{3}$	Drug concentration in plasma (ng/ml)
E2	$Drug_{lumen}^{nM}=\frac{Drug_{lum}}{Mr}*10^{6}$	Drug concentration in kidneys proximal tubules lumen (nM). Mr – molecular weight of compound
E3	*Inhibition of glucose reabsorption mediated by SGLT2* $\begin{array}{l} =SGLT2_{inhibition\_level}^{\%}=\\ =\frac{Drug_{lumen}^{nM}}{IC50_{sglt2}+Drug_{lumen}^{nM}}*100 \end{array}$	Inhibition of glucose reabsorption mediated by SGLT2. SGLT2 inhibition level. Similar expressions are true for description of inhibition level of other transporters with corresponding IC50 values
E4	$SGLT2_{average\_inhibition\_level}^{\%}=\frac{\int_{t1}^{t2}SGLT2_{inhibition\_level}^{\%}}{t2-t1}$	Average inhibition level of SGLT2 during some period of time (from t1 to t2)

### Identification of model parameters

The strategy of model verification is presented in the Supplementary Materials. The dapagliflozin, ipragliflozin, and tofogliflozin models include 19 parameters: 4 physiological, 9 drug specific PK, and 6 drug specific PD. In the right hand side of canagliflozin or empagliflozin models, 17 parameters are included: 4 physiological, 7 drug specific PK, and 6 drug specific PD. The parameter values are specific to each SGLT2 inhibitor model, with the exception of 4 physiological parameters (*V_plasma_, V_lumen_, GFR*, and Q_*urine*_) that have equal values in each model. The physiological parameter values were taken (or calculated) from the literature, as were those for 2 PK parameters (*F* and *fup*) and 6 PD parameters (IC50 for SGLT1–6). The remaining 7 or 5 parameters were fitted against PK data on the dynamics of compounds in plasma and urine. The 95% confidence intervals were calculated for fitted parameters. Parameter values, 95% confidence intervals, and the source of data for their identification are presented in Supplementary Table 1.

### Compartment volumes

Volume of blood plasma was taken from the literature. The volume of the lumen of the kidney's proximal tubules was calculated using experimental data (see Supplementary Materials). In the models that described dapagliflozin, ipragliflozin, and tofogliflozin, distribution volume in peripheral compartments was fitted against PK data. Compartment volumes and the source of identification are presented in Supplementary Table 1.

### Simulations

Model simulations are presented as curves (simulated with optimal parameters values) with shadows (95% confidence bands) or as bars (simulated with optimal parameters values) with error bars (95% confidence bands). The description of simulating the 95% confidence bands is presented in the Supplementary Materials.

## Results

**Figure 2:**
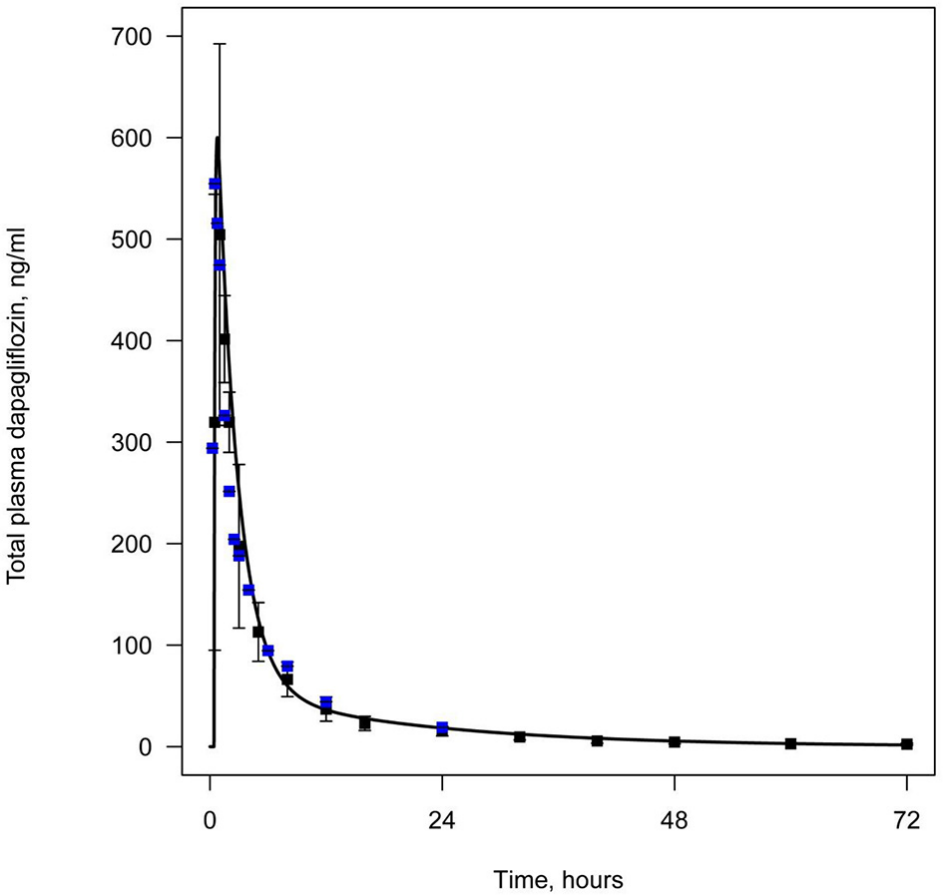
**Example of verification of the dapagliflozin model using plasma data**. Total level of dapagliflozin in plasma following simulation of a single administration of 50 mg. Curve represents model simulation and dots represent experimental data. Colors of dots correspond to different data sources: black—Kasichayanula et al. (2011b), blue—Obermeier et al. (2010).

**Figure 3:**
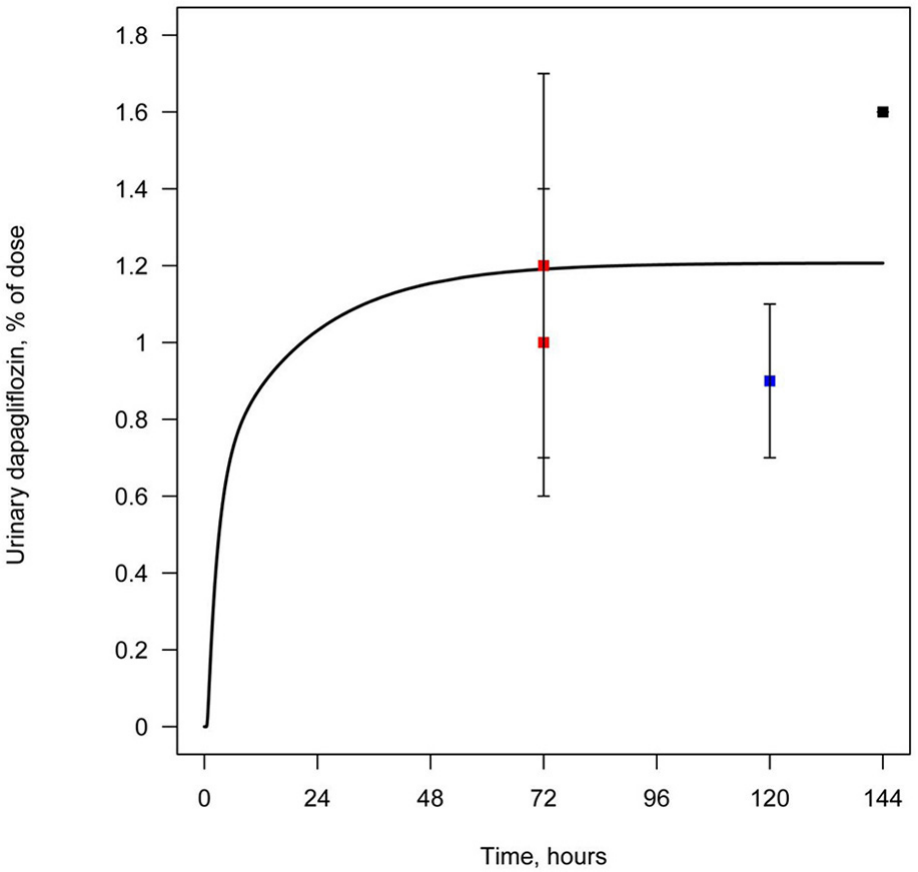
**Example of verification of the dapagliflozin model using urine data**. Cumulative amount of dapagliflozin recovered in urine following simulation of a single administration of 50 mg. Curve represents model simulation and dots represent experimental data. Colors of dots correspond to different data sources: black—Obermeier et al. (2010); blue—Kasichayanula et al. (2011a); red—Kasichayanula et al. (2013).

**Figure 4:**
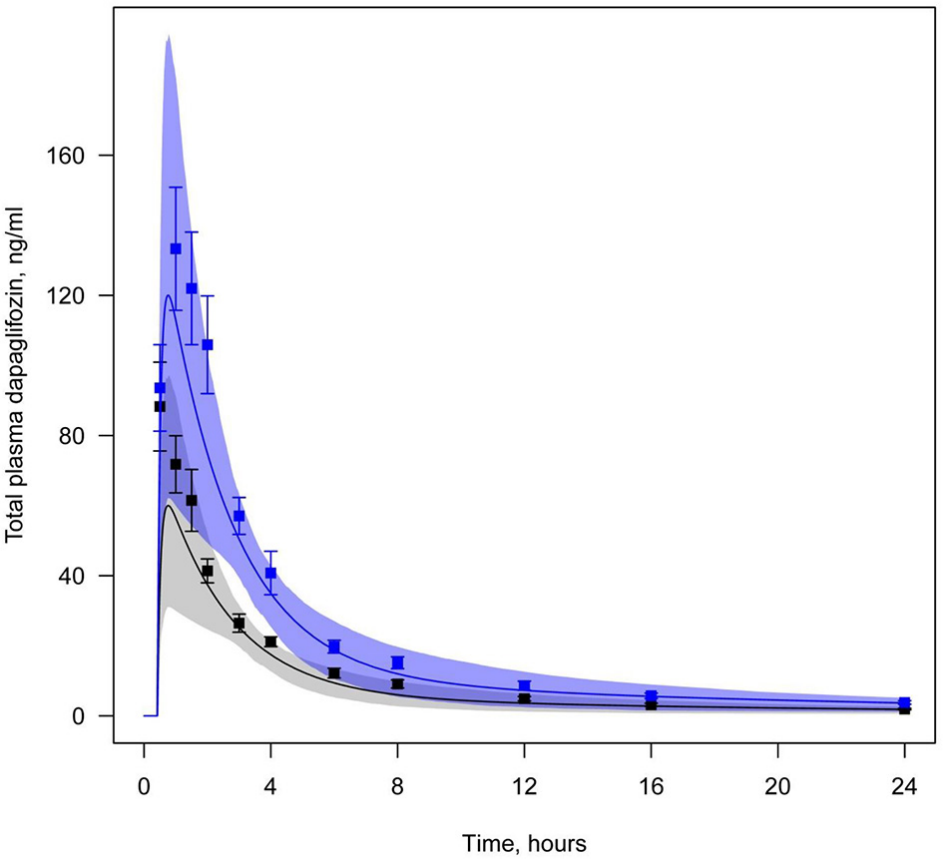
**Example of validation of the dapagliflozin model using plasma data**. Total level of dapagliflozin in plasma following simulation of a single administration, but at different doses (Yang et al., 2013). Curve represents model simulations and dots represent experimental data. Colors of curves and dots correspond to the different doses: black—5 mg, blue—10 mg. Model simulations are presented with 95% confidence bands.

**Figure 5:**
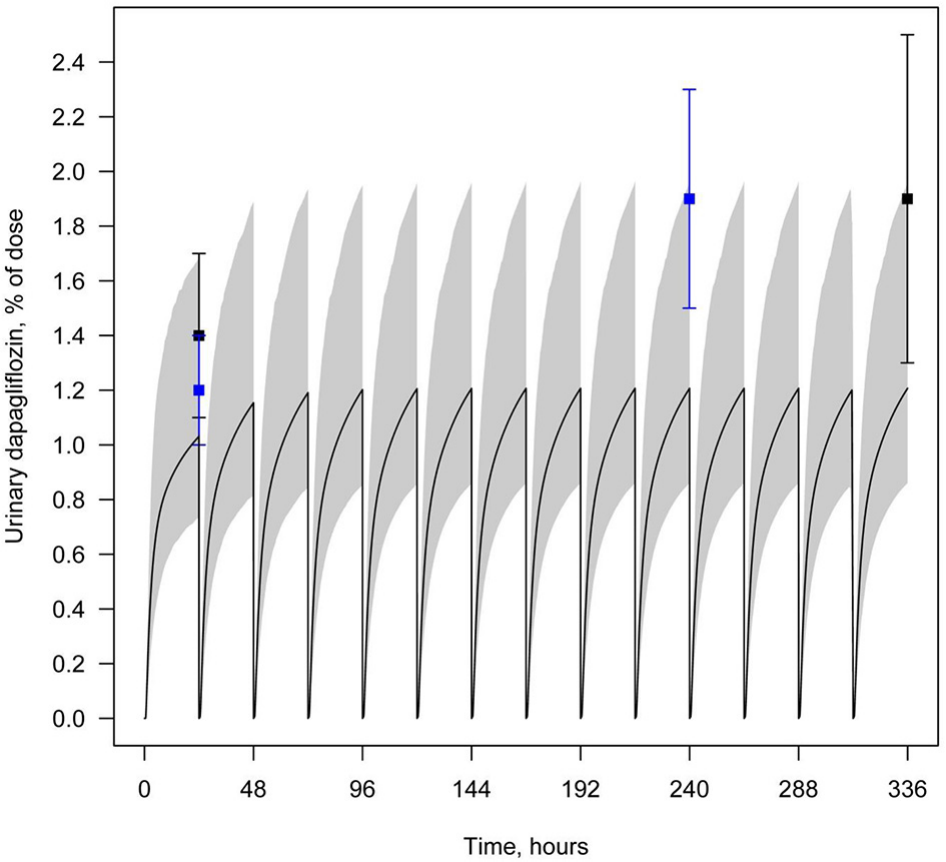
**Example of validation of the dapagliflozin model using urine data**. Amount of dapagliflozin recovered in urine every 24 h following simulation of multiple administrations of 10 mg QD. Curve represents model simulation and dots represent experimental data. Colors of dots correspond to the different data sources: black—Kasichayanula et al. (2011a), blue—Yang et al. (2013). Model simulation is presented with 95% confidence bands.

All data available in the literature was used for model verification and validation (Table 3). Models were calibrated against clinical data obtained in trials where a single dose of SGLT2 inhibitor was administered. By applying the verification strategy described in the Supplementary Materials, we found that: (i) the active secretion of dapagliflozin, canagliflozin, and ipragliflozin from plasma to kidney lumen was equal to zero; (ii) the reabsorption of empagliflozin was equal to zero; and (iii) both active secretion and reabsorption of tofogliflozin were equal to zero. The appropriate choice of model parameters was validated against data obtained from single and multiple dose administration. Figures 2, 3 represent examples of dapagliflozin model calibration using plasma PK and urine recovery data respectively, where data was obtained from single dose trials. Figures 4, 5 also represent examples of dapagliflozin model validation against plasma PK and urine recovery data respectively, but data was obtained from multiple dose trials. The 95% confidence bands captured the variability in clinical data (Figures 4, 5); therefore, we concluded that model validation was of satisfactory quality. Several additional figures demonstrating the quality of verification and validation in dapagliflozin, canagliflozin, ipragliflozin, empagliflozin, and tofogliflozin models are presented in the Supplementary Materials (Supplementary Figures 1–16). These figures demonstrate that the models enable a satisfactory description of plasma PK and recovery of SGLT2 inhibitors in urine.

**Table 3:** **Amount of data used for model verification and validation**.

**Compound**	**Verification**		**Validation**	
	**Number of experimental points**	**Number of articles**	**Number of experimental points**	**Number of articles**
Dapagliflozin	127	9	315	5
Canagliflozin	45	1	79	2
Empagliflozin	158	7	240	5
Ipragliflozin	45	2	210	1
Tofogliflozin	24	2	–	–

### Concentration of SGLT2 inhibitors in the lumen of the kidney's proximal tubules

**Figure 6:**
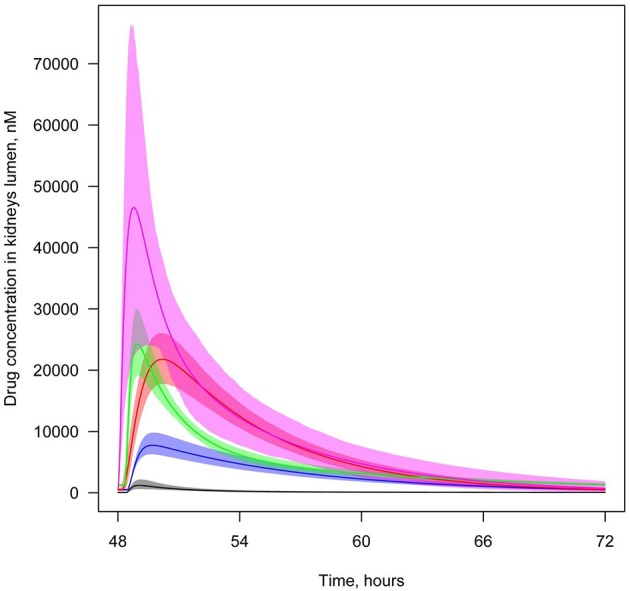
**Concentration of SGLT2 inhibitors in the lumen of the kidney's proximal tubules**. Level of SGLT2 inhibitors in the lumen of the kidney's proximal tubules following simulation of multiple administrations of labeled doses of marketed SGLT2 inhibitors and maximal doses of other SGLT2 inhibitors approved for phase 2/3 studies. Colors of curves correspond to different compounds: black—10 mg QD dapagliflozin; blue—300 mg QD canagliflozin; red—25 mg QD empagliflozin; green—300 mg QD ipragliflozin; pink—40 mg QD tofogliflozin. Model simulations are presented with 95% confidence bands.

To analyze and compare the concentration of drugs in the lumen of the kidney's proximal tubules–the predicted site of inhibition–we simulated time profiles of the drugs resulting from oral administration at various dosages. Figure 6 demonstrates that concentration levels of tofogliflozin, ipragliflozin, and empagliflozin are higher than levels of other inhibitors following administration of marketed SGLT2 inhibitors at labeled doses and non-marketed SGLT2 inhibitors at maximal doses (approved for phase 2/3 studies).

In order to investigate which of five compounds is most likely to accumulate at the potential site of action, we also simulated concentration levels of SGLT2 inhibitors in the lumen of the kidney's proximal tubules following a standardized dosage of the compounds equal to 20 mg. In this scenario, the model predicted that concentrations of empagliflozin and tofogliflozin were higher than the levels of dapagliflozin, canagliflozin, and ipragliflozin (Supplementary Figure 17).

**Figure 7:**
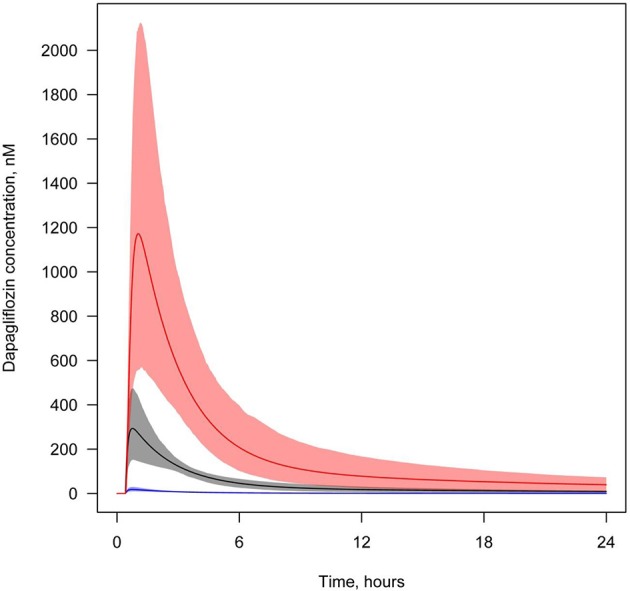
**Concentration of dapagliflozin in plasma and the lumen of the kidney's proximal tubules**. Dapagliflozin levels found in different compartments following simulation of a single administration of 10 mg. Colors of curves correspond to various compartments. Black—total plasma concentration; blue—unbound plasma concentration; red—concentration in lumen. Model simulations are presented with 95% confidence bands.

**Figure 8:**
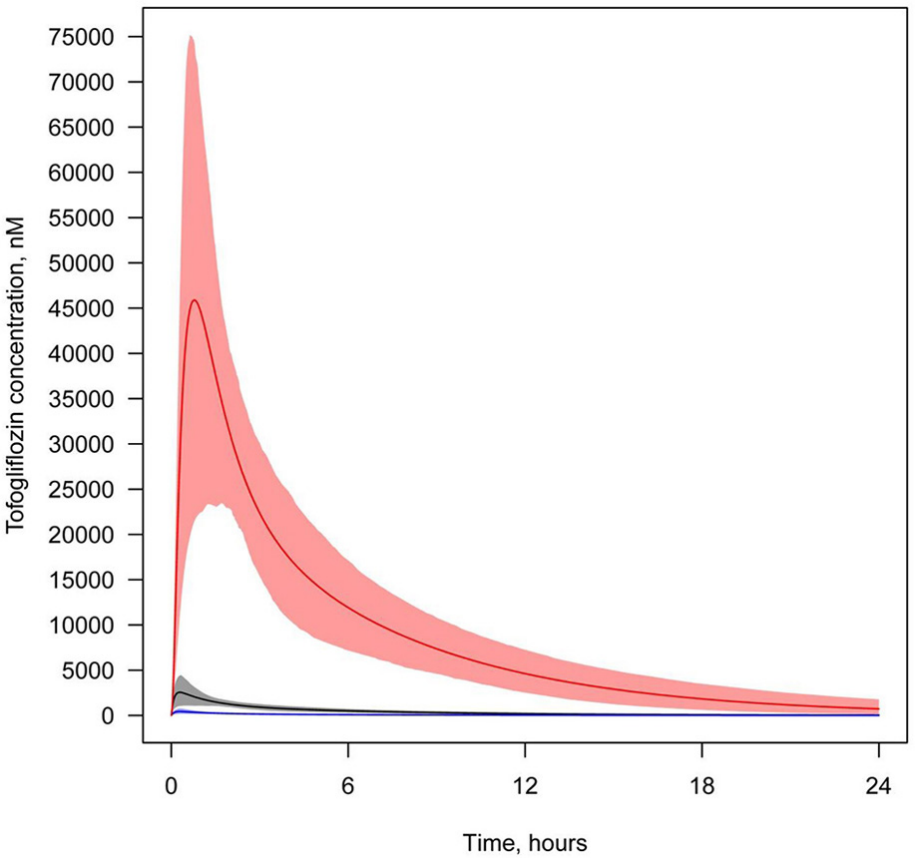
**Concentration of tofogliflozin in plasma and the lumen of the kidney's proximal tubules**. Tofogliflozin levels found in different compartments following simulation of a single administration of 40 mg. Colors of curves correspond to various compartments. Black—total plasma concentration; blue—unbound plasma concentration; red—concentration in lumen. Model simulations are presented with 95% confidence bands.

We also simulated and compared time profiles of SGLT2 inhibitor concentrations in plasma and lumen of the kidney. For all SGLT2 inhibitors, our model predicted that the concentration in the lumen of the kidneys was higher than in plasma (Figures 7, 8, Supplementary Figures 18–20).

### Levels of SGLT2 inhibition during treatment with SGLT2 inhibitors

**Figure 9:**
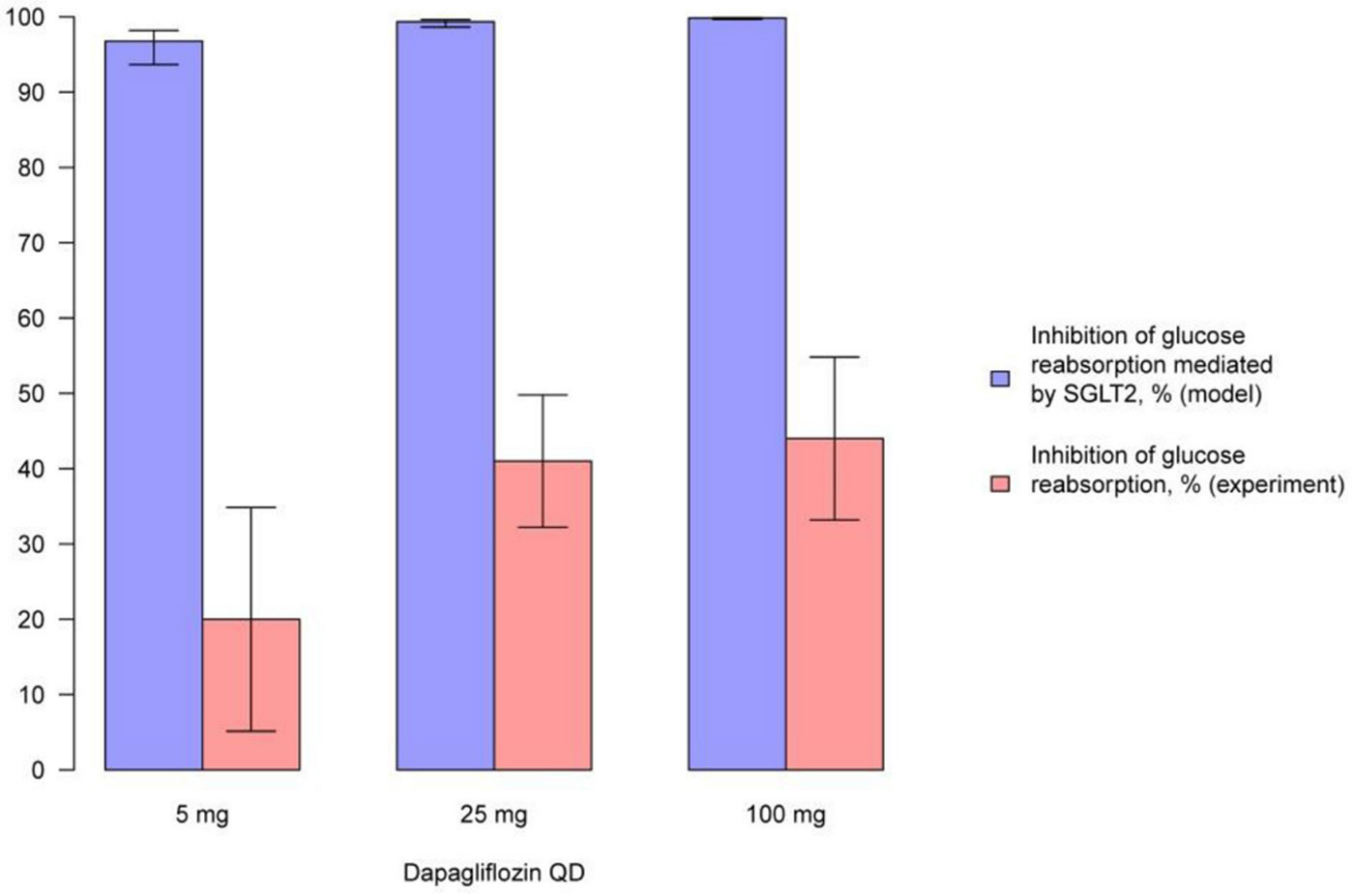
**Comparison of simulated average inhibition of glucose reabsorption mediated by SGLT2 and clinically measured glucose reabsorption inhibition levels during treatment with dapagliflozin**. Comparison of the average inhibition of glucose reabsorption mediated by SGLT2 (simulated in the model) and levels of glucose reabsorption inhibition (measured in experiment) on the 14th day following multiple administrations of different doses of dapagliflozin (Komoroski et al., 2009a). Model simulations are presented with 95% confidence bands.

**Figure 10:**
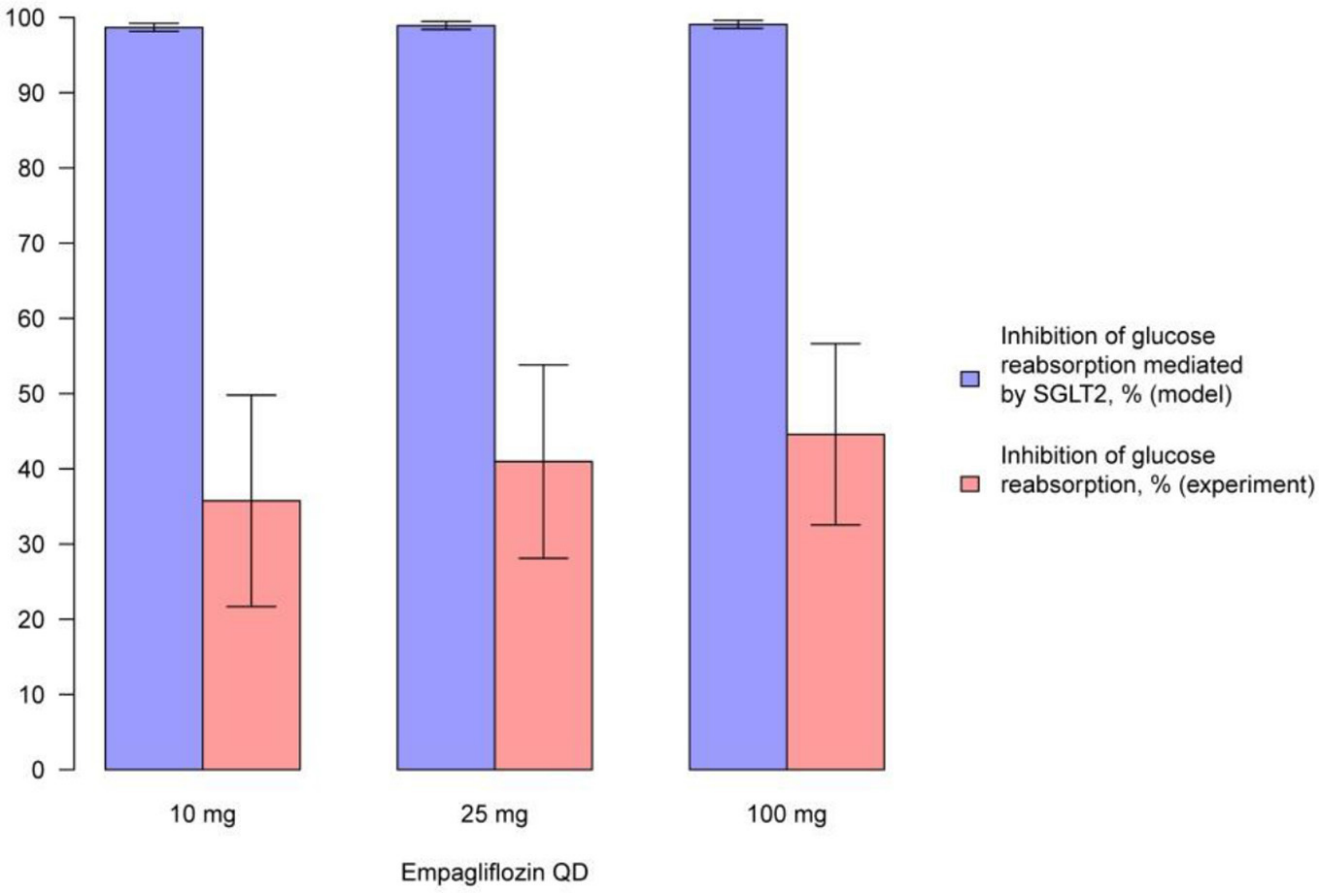
**Comparison of simulated average inhibition of glucose reabsorption mediated by SGLT2 and clinically measured glucose reabsorption inhibition levels during treatment with empagliflozin**. Comparison of the average inhibition of glucose reabsorption mediated by SGLT2 (simulated in the model) and levels of glucose reabsorption inhibition (measured in experiment) on the 1st day after multiple administrations of different doses of empagliflozin (Heise et al., 2013a). Model simulations are presented with 95% confidence bands.

We simulated the average level of inhibition of glucose reabsorption mediated by SGLT2 and compared our findings with experimentally measured levels of glucose reabsorption inhibition following treatment with compounds (Figures 9, 10, Supplementary Figures 21–23). In model simulations, the average level of inhibition of glucose reabsorption mediated by SGLT2 was higher than the level of total glucose reabsorption inhibition, and almost equal to 100%, following treatment with all experimental doses of SGLT2 inhibitors (Figures 9, 10, Supplementary Figures 21–23).

**Figure 11:**
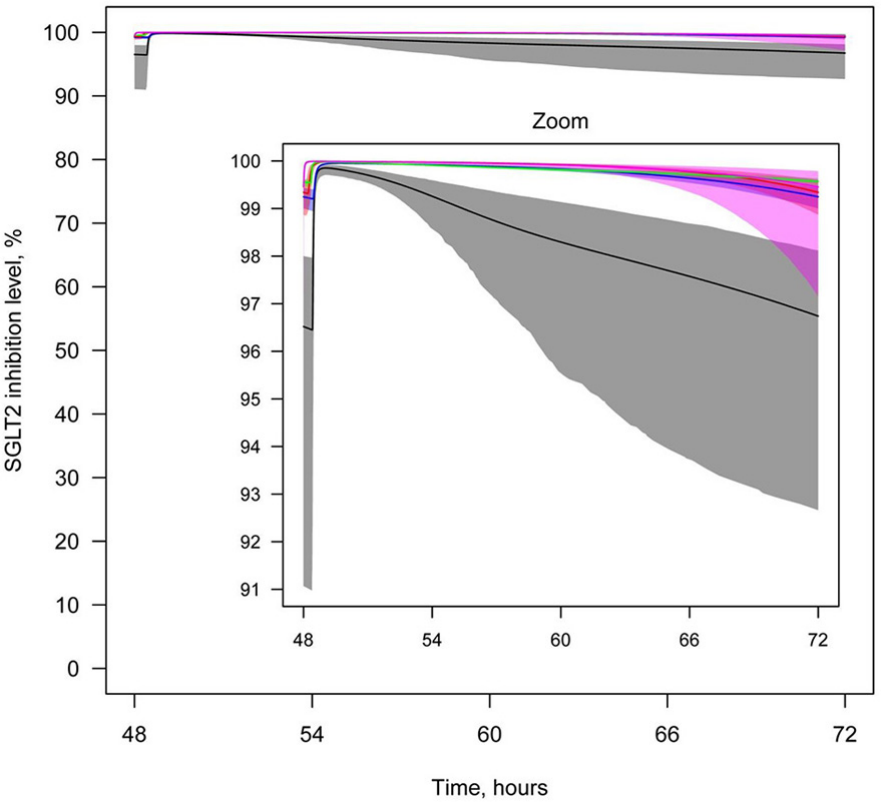
**Levels of SGLT2 inhibition after drug administration**. Levels of SGLT2 inhibition following simulation of multiple administrations of labeled doses of marketed SGLT2 inhibitors and maximal doses of other SGLT2 inhibitors approved for phase 2/3 studies. Colors of curves correspond to different compounds: black—10 mg QD dapagliflozin; blue—300 mg QD canagliflozin; red—25 mg QD empagliflozin; green—300 mg QD ipragliflozin; pink—40 mg QD tofogliflozin. Model simulations are presented with 95% confidence bands.

To compare the efficacies of each compound, the level of SGLT2 inhibition was simulated after administration of equal doses (20 mg) (Supplementary Figure 24), labeled doses of marketed inhibitors, and maximal doses of other inhibitors approved for phase 2/3 studies (Figure 11). For all compounds, administration of equal or labeled/maximal approved doses resulted in almost 100% inhibition of SGLT2. Therefore, the difference in efficacy of the tested SGLT2 inhibitors was not significant.

### Effect of SGLT2 inhibitors on other transporters from the SGLT family

To predict the effect of dapagliflozin, canagliflozin, ipragliflozin, empagliflozin, and tofogliflozin on other members of the SGLT family, the inhibition level of these transporters in the lumen of the kidney's proximal tubules was simulated following administration of labeled doses of marketed inhibitors and maximal doses of other inhibitors approved for phase 2/3 studies. We found that the influence of the inhibitors on other transporters was substantial–approximately 50% or above for particular compounds (Supplementary Figure 25–29). The most effective inhibitor of SGLT1 was canagliflozin (Supplementary Figure 25), while ipragliflozin and tofogliflozin produced the strongest inhibition of SGLT3 (Supplementary Figure 26). While all other SGLT2 inhibitors have a similar effect on SGLT4, dapagliflozin is a weak inhibitor of this co-transporter (Supplementary Figure 27). Ipragliflozin and empagliflozin are the strongest SGLT5 inhibitors (Supplementary Figure 28), while canagliflozin has the strongest effect on SGLT6 (Supplementary Figure 29).

## Discussion

SGLT2 is a promising target for the treatment of T2DM because its inhibition could lower glucose levels without directly influencing insulin resistance or b-cell function. However, in a previous study, administration of SGLT2 inhibitors resulted in only 30–50% inhibition of glucose reabsorption in human subjects even at high doses, which was in contrast to the expected inhibition of 90% based on *in vitro* potency and plasma levels (Liu et al., 2012). In our study, we applied a systems pharmacology modeling approach to test several hypotheses that attempt to explain the discrepancy between the expected and observed levels of glucose reabsorption following administration of SGLT2 inhibitors. We developed models, simulated concentrations of SGLT2 inhibitors in the lumen of the kidney's proximal tubules, and compared the efficacy of these compounds in terms of levels of *in vivo* SGLT2 inhibition.

Models for SGLT2 inhibitors were verified and validated against all available clinical data describing the PK of the compounds in plasma and urine. Similar to the model of Maurer et al. (2011), we introduced a peripheral compartment into our model of dapagliflozin. We also included this compartment for ipragliflozin and tofogliflozin models. Despite the inclusion of a peripheral compartment in a previously published model of empagliflozin (Riggs et al., 2013), we chose a model without peripheral compartment for empagliflozin based on Akaike Information Criterion (AIC) (Akaike, 1974). The AIC we calculated for the empagliflozin model without a peripheral compartment was lower than that of the model with peripheral compartment (343 vs. 345, respectively). Our canagliflozin model was also developed on the basis of one compartmental PK model.

Applying a verification strategy described in the Supplementary Materials, we were able to estimate the models' parameter values for active secretion of the drugs from plasma to urine and reabsorption from urine to plasma. Indeed, we found that the active secretion from plasma to the lumen of the kidneys was equal to zero for dapagliflozin, canagliflozin, and ipragliflozin, but non-zero reabsorption should be taken into account to describe available clinical data. The reabsorption of empagliflozin was equal to zero, but non-zero secretion contributes substantially to the balance of the drug between plasma and urine. Both active secretion and reabsorption of tofogliflozin were equal to zero. Plasma and urine PK data are not sufficient to evaluate the unique values of the rate constants that are responsible for reabsorption and secretion simultaneously. These two parameters are correlated with each other. For tofogliflozin, there is lack of clinical data available to evaluate at least one of these parameters. To understand whether the uncertainty in these parameter values affects the description of clearance in the model, the total systemic and renal clearances measured in clinical trials were compared with those calculated in the model. The total systemic clearance of dapagliflozin was 265 mL/min in the model and 207 mL/min in clinical trials (Boulton et al., 2013). The renal clearance of dapagliflozin was 3.95 mL/min in the model and 3–5 mL/min in clinical trials (Komoroski et al., 2009a,b; Kasichayanula et al., 2011a). The renal clearances of empagliflozin and ipragliflozin in the model were 35 and 1.88 mL/min respectively, while they were 30–37 mL/min for empagliflozin (Heise et al., 2013a,b) and 1–3 mL/min for ipragliflozin (Veltkamp et al., 2011; Zhang et al., 2013) in clinical data. The total systemic clearance of tofogliflozin was equal to 9.9 L/h in the model and 10 L/h in clinical trials (Schwab et al., 2013). The renal clearance of tofogliflozin was equal to 20 mL/min in the model and 25.7 mL/min in clinical trials (Schwab et al., 2013). Therefore, the values produced by our models are similar to experimentally measured values.

One hypothesis proposed by Liu et al. (2012) states that the low efficacy of SGLT2 inhibitors could be explained by the low concentration of compounds at the potential site of action–the lumen of kidney's proximal tubules. Our model suggests that concentrations of SGLT2 inhibitors in the lumen are higher than the corresponding unbound and total concentrations in plasma. This can be explained in terms of reabsorption of water in kidney's proximal tubules (Panchapakesan et al., 2009). A decrease in water volume in the lumen leads to an increase in the concentration of the compounds in lumen than in the plasma. In the model, this is reflected by the ratio of glomerular filtration and urine excretion (urine formation) rates. Thus, we conclude that the concentration of compounds in the lumen of the kidney's proximal tubules is actually relatively high, and we reject the hypothesis that the low efficacy of SGLT2 inhibitors is caused by their low concentration at the site of action. Our model predicts that concentrations of empagliflozin and tofogliflozin in kidney lumen are higher than concentrations of dapagliflozin, canagliflozin, and ipragliflozin following administration of equal doses. This prediction is supported by data obtained from clinical trials, which indicates that approximately 20% of the empagliflozin and tofogliflozin dose is recovered in urine (Brand et al., 2012; Zell et al., 2013) in comparison to about 1% for the other SGLT2 inhibitors (Kasichayanula et al., 2011a; Devineni et al., 2013; Zhang et al., 2013).

The model shows that administration of labeled doses of marketed inhibitors (dapagliflozin and canagliflozin) and of maximal doses approved for phase 2/3 studies of tofogliflozin, ipragliflozin, and empagliflozin leads to almost complete inhibition of SGLT2 *in vivo*. The average inhibition level of glucose reabsorption mediated by SGLT2 (the average SGLT2 inhibition level) predicted by the model is higher than glucose reabsorption inhibition level measured in clinical trials, and almost equal to 100%. Thus, if the lumen of the kidney's proximal tubules is indeed the site of action of SGLT2 inhibitors, and SGLT2 is the main transporter that facilitates reabsorption of glucose, then there is a contradiction between the simulations produced by the model and the clinical data. This contradiction leads us to support two hypotheses proposed by Liu et al. (2012). Firstly, that the potential site of action of SGLT2 inhibitors is not in the lumen of the kidney's proximal tubules, but in the proximal tubule cells. Secondly, that there are other transporters that could facilitate glucose reabsorption under the conditions of SGLT2 inhibition. Both hypotheses appear reasonable within the framework of our current model. To determine which of these two hypotheses is true, further modeling supported by additional experimental data is required.

The model was applied to compare the efficacy of different SGLT2 inhibitors in respect of inhibiting other transporters in the SGLT family that are expressed in kidneys. Simulating the administration of equal doses of compounds (20 mg), labeled doses of marketed inhibitors, and maximal doses of other inhibitors approved for phase 2/3 studies showed that levels of SGLT2 inhibition are similar for all compounds and almost equal to 100%. This is caused by high concentrations of SGLT2 inhibitors in the lumen of the kidneys and low *in vitro* IC50 values. The effect of SGLT2 inhibitors on other transporters is also rather strong because of its high concentration in kidney's lumen. Canagliflozin is the strongest SGLT1 and SGLT6 inhibitor, and this is caused by canagliflozin's low IC50 (the lowest of all the SGLT2 inhibitors considered here). The most effective SGLT3 inhibitors are ipragliflozin and tofogliflozin because of their high *in vitro* potencies. All compounds have similar effects on SGLT4, except dapagliflozin. Dapagliflozin has the weakest influence on SGLT4 because of its low concentration in the kidney's lumen. Ipragliflozin and empagliflozin are the strongest SGLT5 inhibitors because of their low IC50 of for SGLT5, and the high concentration of ipragliflozin in the lumen.

In conclusions, we have developed a systems pharmacology model for SGLT2 inhibitors that enables the estimation of its concentration in the lumen of the kidney's proximal tubules (the potential site of SGLT2 action) and the prediction of SGLT2 inhibition levels during treatment in humans. We have shown that the concentration of SGLT2 inhibitors in the lumen of the proximal tubules is high, and that the level of SGLT2 inhibition during treatment in humans is almost 100%. Based on the results of our model, two explanations for the observed low efficacy of SGLT2 inhibitors were supported: (1) the site of action of SGLT2 inhibitors is not in the lumen of the kidney's proximal tubules, but elsewhere (e.g., the kidneys proximal tubule cells); and (2) there are other transporters that could facilitate glucose reabsorption under the conditions of SGLT2 inhibition (e.g., other transporters of SGLT family). It was found that all SGLT2 inhibitors have similar efficacy.

### Conflict of interest statement

The authors declare that the research was conducted in the absence of any commercial or financial relationships that could be construed as a potential conflict of interest.
