# Supplementary material for: Analysis of the efficacy of SGLT2 inhibitors using semi-mechanistic model
Source: Front Pharmacol. 2014 Oct 13;5:218. doi: 10.3389/fphar.2014.00218 (PMC4195280; doi:10.3389/fphar.2014.00218)
Supplement: Supplementary file 1 [file DataSheet1.DOCX]

***Supplementary Material***

**Analysis of efficacy of SGLT2 inhibitors using semi-mechanistic model**

Oleg Demin Jr^1*^, Tatiana Yakovleva^1^, Dmitry Kolobkov^2^, Oleg Demin^2^

^1^Laboratory Alpha, Institute for Systems Biology Moscow, Moscow, Russia

^2^Institute for Systems Biology Moscow, Moscow, Russia

^*^Corresponding author: Oleg Demin Jr

Institute for Systems Biology Moscow

Laboratory Alpha

Nauchny proezd, 20, bldg 2, Technopark Slava

117246, Moscow, Russia

[demin_jr@insysbio.ru](mailto:demin_jr@insysbio.ru)

# Supplementary Data

## Models verification strategy

The following steps were performed during model verification for each compound:

1. Simple one- and two-compartmental models without detailed description of renal clearance were verified against plasma PK data only. If two compartmental model does not lead to improvement of fitting, the one compartmental model was chosen. As a result, the parameters of absorption (rate constant and delay) were evaluated and fixed.
2. The detailed description of renal clearance was added to chosen model. The secretion and reabsorption were equal to zero. The model was verified against data on plasma and urine simultaneously. If the urine data was described satisfactory, the values of secretion and reabsorption were fixed as zero in final model. If the data were overestimated, the constant describing reabsorption was added in the fitting procedure and evaluated and secretion parameter was equal to zero in final model. If the data were underestimated, the constant describing secretion was added in the fitting procedure and evaluated and reabsorption parameter was equal to zero in final model.

## Kidney proximal tubules lumen volume calculation

Volume of proximal tubules lumen for each nephron was calculated using literature data (Beresford, 2000):

V = 3.14 * Radius^2^ * Length = 3.14 * (25 * 10^-6^)^2^ m^2^ * 14.5 * 10-3 m = 28456 * 10^-12^ dm^3^ = 28.5 * 10^-9^ L

It is known that there 900 000 nephron (Hughson et al., 2003) in one kidney and only one third of them is working (Kurepina, 2003). So, there are 300 000 of working nephrons in one kidney. Thus, total volume of proximal tubules lumen for all working nephrons in two kidneys is:

V = 2 * 300 000 * 28.5 * 10^-9^ L = 0.0171 L.

## Excretion to urine flux (urine formation) calculation

It is known that volume of urine during 24 hours is 1.32 liters for healthy subjects (Borghi et al., 1996). Thus, the urine formation flux is:

Q_urine = 1.32 L / 24 hours = 0.055 L/ hour

## Confidence bands simulation

Confidence bands were simulated as follows. First, 1000 points in parameter space were generated. For all fitted parameters normal distribution was assumed, with mean and standard deviation obtained from parameters optimal fitted values and bounds of 95% confidence intervals. Second, 1000 curves were simulated for all 1000 parameter sets. Finally, at each point, confidence bounds were taken so that they include 95% of 1000 curves values. These bounds constitute the confidence band for a simulation curve.

# Supplementary Figures and Tables

## Supplementary Tables

**Supplementary Table 1 | Models parameters**

| Parameter | Description | Value | Left 95% CI* | Right 95% CI | Dimension | Method of identification |
| --- | --- | --- | --- | --- | --- | --- |
| Dapagliflozin parameters | | | | | | |
| *F* | Bioavailability | 0.78 | - | - | - | TAK* (Boulton et al., 2013) |
| $k_{abs}$ | Absorption in intestine | 0.47 | 036 | 0.72 | 1/hour | FIT* (Suppl. Fig.1,2) |
| ${lag}_{abs}$ | Lag (delay) in absorption | 0.418 | 0.364 | 0.453 | hour | FIT (Suppl. Fig.1,2) |
| $k_{deg}$ | Degradation in plasma | 88.51 | 87.53 | 98.3 | 1/hour | FIT (Suppl. Fig.1,2) |
| $Q_{prf}$ | Exchange between central and peripheral compartments | 175 | 114 | 300 | L/hour | FIT (Suppl. Fig.1,2) |
| ${Vd}_{prf}$ | Distribution volume (peripheral compartment) | 2050 | 1590 | 2710 | L | FIT (Suppl. Fig.1,2) |
| *fup* | Fraction unbound in plasma | 0.062 | - | - | - | TAK (Komoroski et al., 2009a; Obermeier et al., 2010; Kasichayanula et al., 2011a) |
| $k_{sec}$ | Secretion from plasma to lumen | 0 | - | - | 1/hour | FIX* |
| $k_{reab}$ | Reabsorption from lumen to plasma | 2.8 | 1.3 | 5.5 | 1/hour | FIT (Suppl. Fig.1,2) |
| ${IC50}_{sglt1}$ | IC50 for SGLT1 | 937 | - | - | nM | TAK (Suzuki et al., 2012; Tahara et al., 2012; Ohtake et al., 2012; Grempler et al., 2012; Meng et al., 2008; Goodwin et al., 2009) |
| ${IC50}_{sglt2}$ | IC50 for SGLT2 | 1.83 | - | - | nM | TAK (Suzuki et al., 2012; Tahara et al., 2012; Ohtake et al., 2012; Grempler et al., 2012; Meng et al., 2008; Goodwin et al., 2009) |
| ${IC50}_{sglt3}$ | IC50 for SGLT3 | 190000 | - | - | nM | TAK (Suzuki et al., 2012) |
| ${IC50}_{sglt4}$ | IC50 for SGLT4 | 6050 | - | - | nM | TAK (Suzuki et al., 2012; Grempler et al., 2012) |
| ${IC50}_{sglt5}$ | IC50 for SGLT5 | 515 | - | - | nM | TAK (Suzuki et al., 2012; Grempler et al., 2012) |
| ${IC50}_{sglt6}$ | IC50 for SGLT6 | 1300 | - | - | nM | TAK (Suzuki et al., 2012; Grempler et al., 2012) |
| *Mr* | Molecular weight | 408.9 | - | - | g/mol | http://pubchem.ncbi.nlm.nih.gov/rest/chemical/9887712 |
| Canagliflozin parameters | | | | | | |
| *F* | Bioavailability | 0.65 | - | - | - | TAK (Invokana Product Information) |
| $k_{abs}$ | Absorption in intestine | 0.124 | 0.112 | 0.134 | 1/hour | FIT (Suppl. Fig.5,6) |
| ${lag}_{abs}$ | Lag (delay) in absorption | 0.453 | 0.439 | 464 | hour | FIT (Suppl. Fig.5,6) |
| $k_{deg}$ | Degradation in plasma | 170 | 160 | 182 | 1/hour | FIT (Suppl. Fig.5,6) |
| *fup* | Fraction unbound in plasma | 0.02 | - | - | - | TAK (Invokana Product Information) |
| $k_{sec}$ | Secretion from plasma to lumen | 0 | - | - | 1/hour | FIX |
| $k_{reab}$ | Reabsorption from lumen to plasma | 2.44 | 1.6 | 3.6 | 1/hour | FIT (Suppl. Fig.5,6) |
| ${IC50}_{sglt1}$ | IC50 for SGLT1 | 637 | - | - | nM | TAK (Suzuki et al., 2012; Grempler et al., 2012; Nomura et al., 2010) |
| ${IC50}_{sglt2}$ | IC50 for SGLT2 | 3.867 | - | - | nM | TAK (Suzuki et al., 2012; Grempler et al., 2012; Nomura et al., 2010) |
| ${IC50}_{sglt3}$ | IC50 for SGLT3 | 52000 | - | - | nM | TAK (Suzuki et al., 2012) |
| ${IC50}_{sglt4}$ | IC50 for SGLT4 | 5350 | - | - | nM | TAK (Suzuki et al., 2012; Grempler et al., 2012) |
| ${IC50}_{sglt5}$ | IC50 for SGLT5 | 940 | - | - | nM | TAK (Suzuki et al., 2012; Grempler et al., 2012) |
| ${IC50}_{sglt6}$ | IC50 for SGLT6 | 220 | - | - | nM | TAK (Suzuki et al., 2012; Grempler et al., 2012) |
| *Mr* | Molecular weight | 444.5 | - | - | g/mol | http://pubchem.ncbi.nlm.nih.gov/rest/chemical/24812758 |
| Empagliflozin parameters | | | | | | |
| *F* | Bioavailability (Apparent) | 0.332 | - | - | - | Taken as for rats from Grempler et al., 2012 |
| $k_{abs}$ | Absorption in intestine | 0.178 | 0.156 | 0.206 | 1/hour | FIT (Suppl. Fig.8,9) |
| ${lag}_{abs}$ | Lag (delay) in absorption | 0.21 | 0.03 | 0.21 | hour | FIT (Suppl. Fig.8,9) |
| $k_{deg}$ | Degradation in plasma | 5.37 | 4.4 | 6.5 | 1/hour | FIT (Suppl. Fig.8,9) |
| *fup* | Fraction unbound in plasma | 0.1485 | - | - | - | TAK (Macha et al., 2014a; Macha et al., 2014b) |
| $k_{sec}$ | Secretion from plasma to lumen | 2.44 | 1.68 | 3.44 | 1/hour | FIT (Suppl. Fig.8,9) |
| $k_{reab}$ | Reabsorption from lumen to plasma | 0 | - | - | 1/hour | FIX |
| ${IC50}_{sglt1}$ | IC50 for SGLT1 | 4700 | - | - | nM | TAK (Suzuki et al., 2012; Grempler et al., 2012) |
| ${IC50}_{sglt2}$ | IC50 for SGLT2 | 3.35 | - | - | nM | TAK (Suzuki et al., 2012; Grempler et al., 2012) |
| ${IC50}_{sglt3}$ | IC50 for SGLT3 | 62000 | - | - | nM | TAK (Suzuki et al., 2012) |
| ${IC50}_{sglt4}$ | IC50 for SGLT4 | 6600 | - | - | nM | TAK (Suzuki et al., 2012; Grempler et al., 2012) |
| ${IC50}_{sglt5}$ | IC50 for SGLT5 | 605 | - | - | nM | TAK (Suzuki et al., 2012; Grempler et al., 2012) |
| ${IC50}_{sglt6}$ | IC50 for SGLT6 | 1550 | - | - | nM | TAK (Suzuki et al., 2012) |
| *Mr* | Molecular weight | 450.9 | - | - | g/mol | http://pubchem.ncbi.nlm.nih.gov/rest/chemical/11949646 |
| Ipragliflozin parameters | | | | | | |
| *F* | Bioavailability | 0.9 | - | - | - | TAK (Zhang et al., 2013) |
| $k_{abs}$ | Absorption in intestine | 0.51 | 0.419 | 0.63 | 1/hour | FIT (Suppl. Fig.12,13) |
| ${lag}_{abs}$ | Lag (delay) in absorption | 0.335 | 0.22 | 0.389 | hour | FIT (Suppl. Fig.12,13) |
| $k_{deg}$ | Degradation in plasma | 122.9 | 116.6 | 128 | 1/hour | FIT (Suppl. Fig.12,13) |
| $Q_{prf}$ | Exchange between central and peripheral compartments | 413 | 330 | 520 | L/hour | FIT (Suppl. Fig.12,13) |
| ${Vd}_{prf}$ | Distribution volume (peripheral compartment) | 2610 | 2340 | 3030 | L | FIT (Suppl. Fig.12,13) |
| *fup* | Fraction unbound in plasma | 0.032 | - | - | - | TAK (Zhang et al., 2013) |
| $k_{sec}$ | Secretion from plasma to lumen | 0 | - | - | 1/hour | FIX |
| $k_{reab}$ | Reabsorption from lumen to plasma | 3.36 | 2.99 | 3.79 | 1/hour | FIT (Suppl. Fig.12,13) |
| ${IC50}_{sglt1}$ | IC50 for SGLT1 | 1904 | - | - | nM | TAK (Suzuki et al., 2012; Imamura et al., 2012; Tahara et al., 2012; Grempler et al., 2012) |
| ${IC50}_{sglt2}$ | IC50 for SGLT2 | 5.72 | - | - | nM | TAK (Suzuki et al., 2012; Imamura et al., 2012; Tahara et al., 2012; Grempler et al., 2012) |
| ${IC50}_{sglt3}$ | IC50 for SGLT3 | 7700 | - | - | nM | TAK (Suzuki et al., 2012) |
| ${IC50}_{sglt4}$ | IC50 for SGLT4 | 10250 | - | - | nM | TAK (Suzuki et al., 2012; Grempler et al., 2012) |
| ${IC50}_{sglt5}$ | IC50 for SGLT5 | 414 | - | - | nM | TAK (Suzuki et al., 2012; Grempler et al., 2012) |
| ${IC50}_{sglt6}$ | IC50 for SGLT6 | 5650 | - | - | nM | TAK (Suzuki et al., 2012; Grempler et al., 2012) |
| *Mr* | Molecular weight | 404.5 | - | - | g/mol | http://pubchem.ncbi.nlm.nih.gov/rest/chemical/10453870 |
| Tofogliflozin parameters | | | | | | |
| *F* | Bioavailability | 0.975 | - | - | - | TAK (Schwab et al., 2013) |
| ${lag}_{abs}$ | Lag (delay) in absorption | 0 | - | - | hour | FIT (Suppl. Fig.15,16) |
| $k_{abs}$ | Absorption in intestine | 0.93 | 0.61 | 1.51 | 1/hour | FIT (Suppl. Fig.15,16) |
| $k_{deg}$ | Degradation in plasma | 18.1 | 15.4 | 22.3 | 1/hour | FIT (Suppl. Fig.15,16) |
| $Q_{prf}$ | Exchange between central and peripheral compartments | 112 | 65 | 184 | L/hour | FIT (Suppl. Fig.15,16) |
| ${Vd}_{prf}$ | Distribution volume (peripheral compartment) | 237 | 194 | 327 | L | FIT (Suppl. Fig.15,16) |
| *fup* | Fraction unbound in plasma | 0.17 | - | - | - | TAK (Schwab et al., 2013) |
| $k_{sec}$ | Secretion from plasma to lumen | 0 | - | - | 1/hour | FIX |
| $k_{reab}$ | Reabsorption from lumen to plasma | 0 | - | - | 1/hour | FIX |
| ${IC50}_{sglt1}$ | IC50 for SGLT1 | 7781 | - | - | nM | TAK (Suzuki et al., 2012; Ohtake et al., 2012; Grempler et al., 2012) |
| ${IC50}_{sglt2}$ | IC50 for SGLT2 | 4.067 | - | - | nM | TAK (Suzuki et al., 2012; Ohtake et al., 2012; Grempler et al., 2012) |
| ${IC50}_{sglt3}$ | IC50 for SGLT3 | 19000 | - | - | nM | TAK (Suzuki et al., 2012) |
| ${IC50}_{sglt4}$ | IC50 for SGLT4 | 7750 | - | - | nM | TAK (Suzuki et al., 2012; Grempler et al., 2012) |
| ${IC50}_{sglt5}$ | IC50 for SGLT5 | 1770 | - | - | nM | TAK (Suzuki et al., 2012; Grempler et al., 2012) |
| ${IC50}_{sglt6}$ | IC50 for SGLT6 | 6200 | - | - | nM | TAK (Suzuki et al., 2012) |
| *Mr* | Molecular weight | 386 | - | - | g/mol | http://pubchem.ncbi.nlm.nih.gov/rest/chemical/46908929 |
| General parameters for all compounds | | | | | | |
| GFR | Glomerular filtration rate | 7.2 | - | - | L/hour | Correspond to normal GFR = 120 ml/min |
| $Q_{urine}$ | Excretion from lumen to urine (urine formation) | 0.055 | - | - | 1/hour | Calculated based on Borghi et al., 1996 |
| $V_{plasma}$ | Plasma volume | 2.75 | - | - | L | TAK (Retzlaff et al., 1969) |
| $V_{lumen}$ | Kidneys proximal tubules lumen volume | 0.0171 | - | - | L | Calculated based on Hughson et al., 2003; Beresford, 2000; Kurepina, 2003 |

*CI – Confidential Interval

*TAK – Taken from

*FIT – Fitted against PK data on plasma and urine

*FIX – Fixed

## Supplementary Figures

### Dapagliflozin

#### Verification


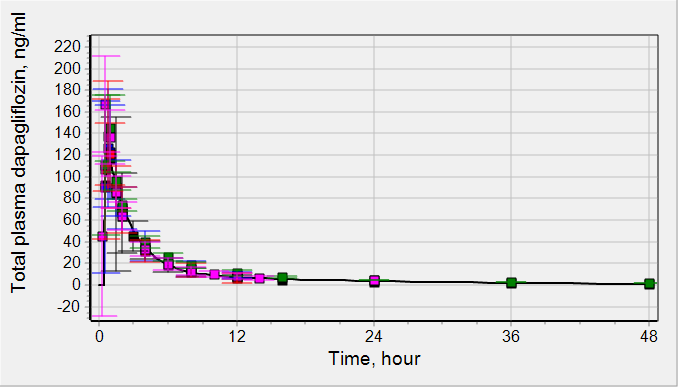

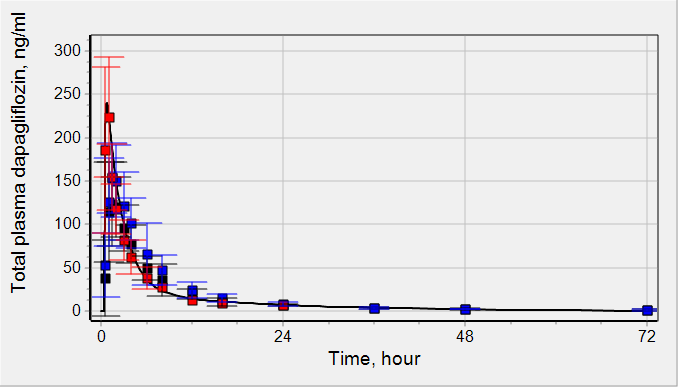


A

B


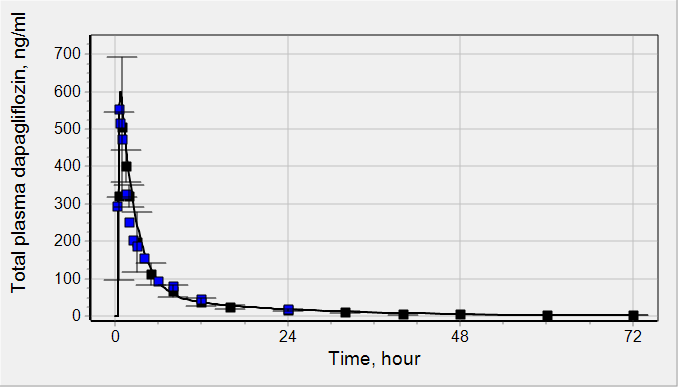


C

**Supplementary Figure 1 | Dapagliflozin model verification against plasma data**

Total plasma dapagliflozin after single administration of different doses was simulated. Colors of points correspond to the different sources. (A) Administration of 10 mg of dapagliflozin: black – Kasichayanula et al., 2011a; blue and red – Kasichayanula et al., 2013a; green – Boulton et al., 2013; pink – Imamura et al., 2013. (B) Administration of 20 mg of dapagliflozin: black and blue – Kasichayanula et al., 2011b; red – Kasichayanula et al., 2012. (C) Administration of 50 mg of dapagliflozin: black – Kasichayanula et al., 2011b; blue – Obermeier et al., 2010.

**
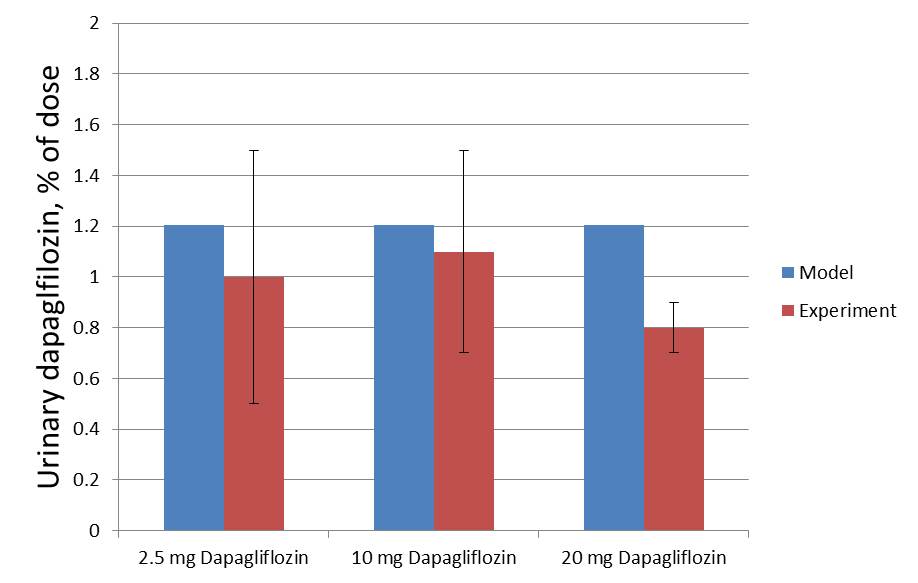

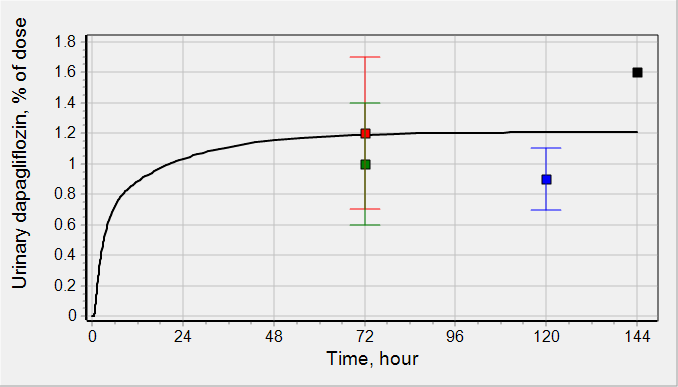
**

B

A

**Supplementary Figure 2 | Dapagliflozin model verification against urine data**

Cumulative amount of dapagliflozin recovered in urine after single administration of different doses was simulated. (A) Cumulative amount of dapagliflozin recovered in urine during 120 hours after single administration of 2.5, 10 and 20 mg of dapaglflozin (Kasichayanula et al., 2011c). (B) Cumulative amount of dapagliflozin recovered in urine during different periods after single administration of 50 mg. Colors of points correspond to the different sources: black – Obermeier et al., 2010; blue – Kasichayanula et al., 2011c; red and green – Kasichayanula et al., 2013b.

#### Validation


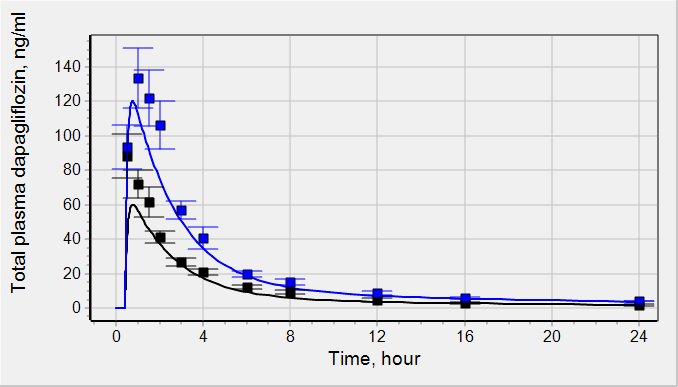

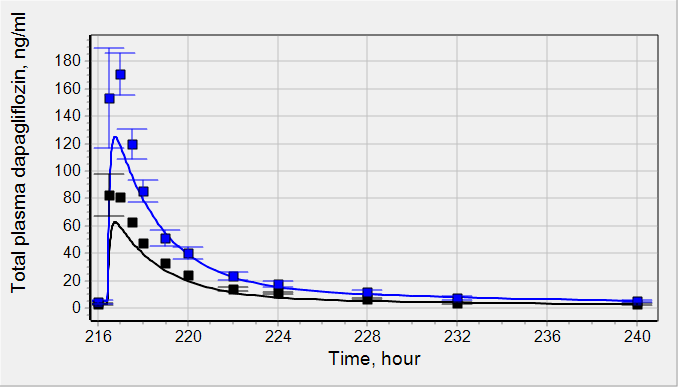


B

A


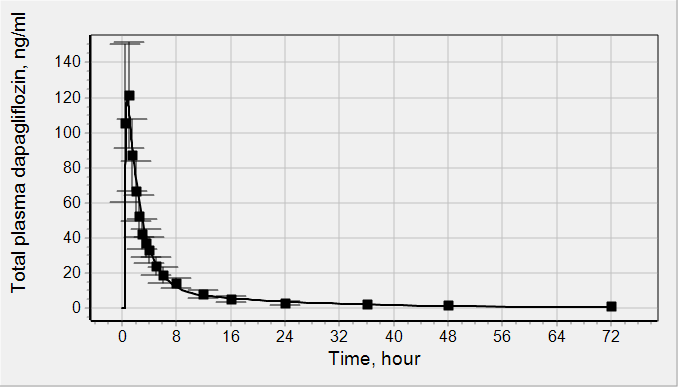

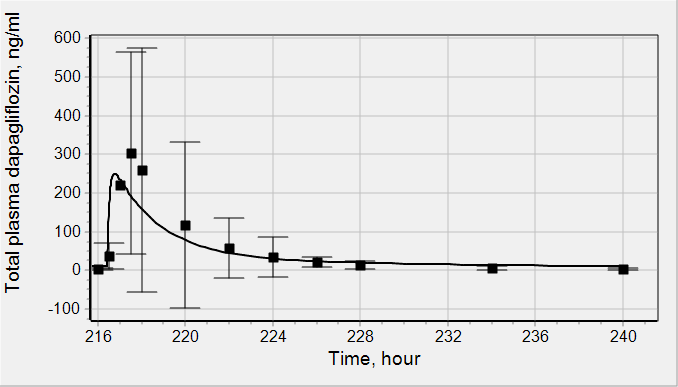


C

D


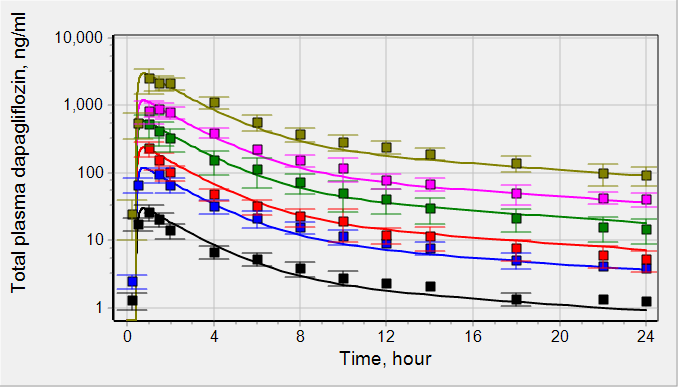

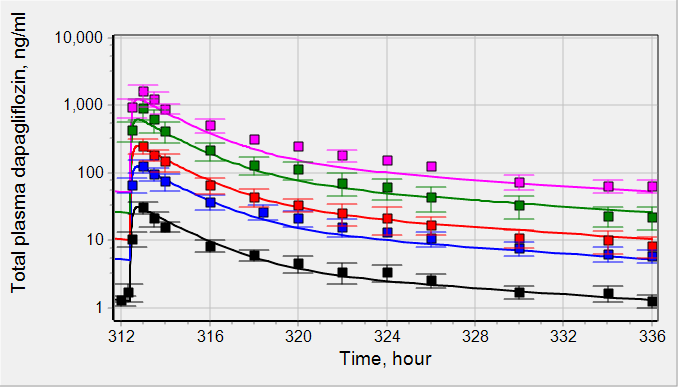


F

E


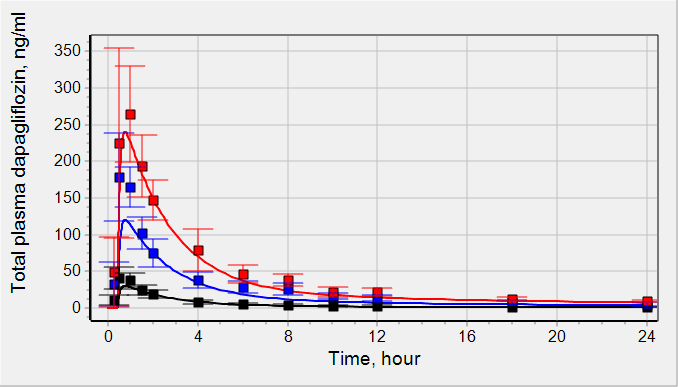

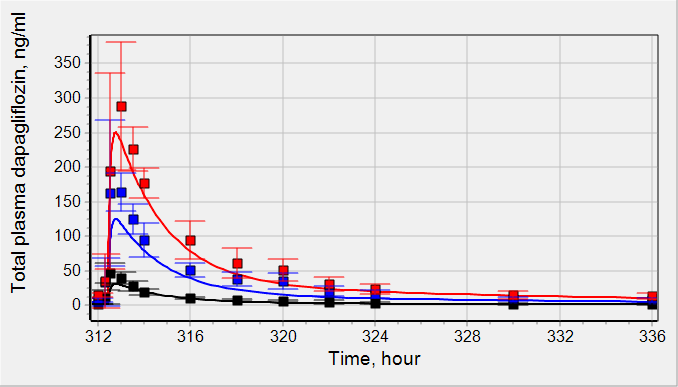


H

G

**Supplementary Figure 3 | Dapagliflozin model validation against plasma data**

Total plasma dapagliflozin after administrations of different doses was simulated. (A) Total plasma dapagliflozin after single administration of different doses (Yang et al., 2013). Colors of points correspond to the different doses: black – 5 mg, blue – 10 mg. (B) Total plasma dapagliflozin on 10^th^ day after multiple administrations of different doses (Yang et al., 2013). Colors of points correspond to the different doses: black – 5 mg QD, blue – 10 mg QD. (C) Total plasma dapagliflozin after single administration of 10mg under fasted conditions (Kasichayanula et al., 2011d). (D) Total plasma dapagliflozin on 10^th^ day after multiple administrations of 20mg QD (Kasichayanula et al., 2013b). (E) Total plasma dapagliflozin after single administration of different doses (Komoroski et al., 2009a). Colors of points correspond to the different doses: black –2.5 mg; blue – 10 mg; red – 20 mg; green – 50 mg; pink – 100 mg; brown – 250 mg. (F) Total plasma dapagliflozin on 14^th^ day after multiple administrations of different doses (Komoroski et al., 2009a). Colors of points correspond to the different doses: black –2.5 mg QD; blue – 10 mg QD; red – 20 mg QD; green – 50 mg QD; pink – 100 mg QD. (G) Total plasma dapagliflozin after single administration of different doses (Kasichayanula et al., 2011c). Colors of points correspond to the different doses: black –2.5 mg; blue – 10 mg; red – 20 mg. (H) Total plasma dapagliflozin on 14^th^ day after multiple administrations of different doses (Kasichayanula et al., 2011c). Colors of points correspond to the different doses: black –2.5 mg QD; blue – 10 mg QD; red – 20 mg QD.


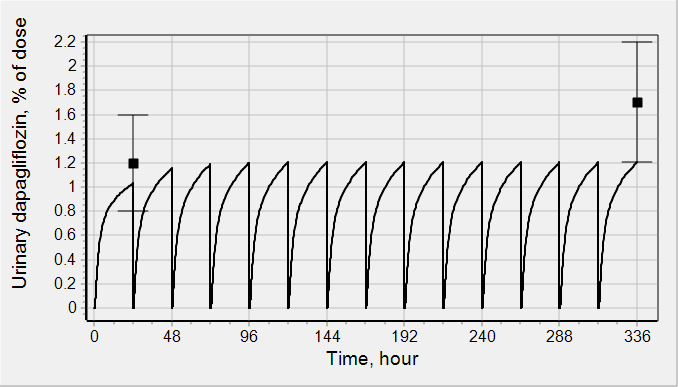

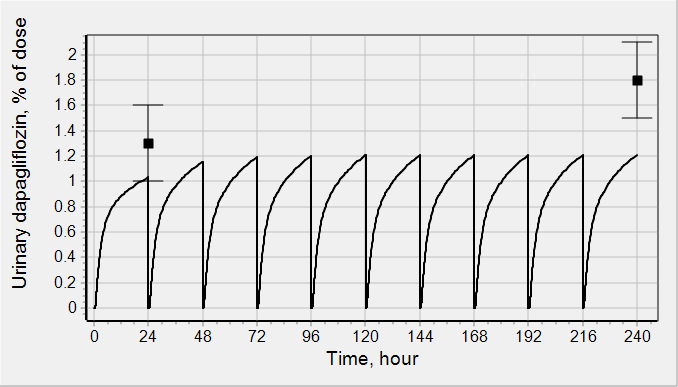


B

A


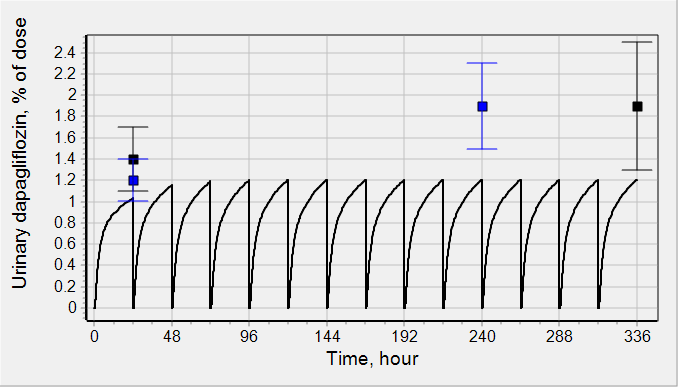

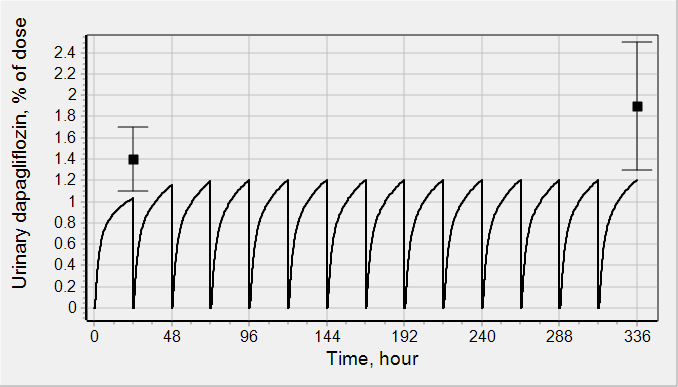


D

C

**Supplementary Figure 4 | Dapagliflozin model validation against urine data**

Amount of dapagliflozin recovered in urine every 24 hours after multiple administrations of different doses was simulated. (A) Administration of 2.5 mg QD (Kasichayanula et al., 2011c). (B) Administration of 5 mg QD (Yang et al., 2013). (C) Administration of 10 mg QD. Colors of points correspond to the different sources: black – Kasichayanula et al., 2011c; blue – Yang et al., 2013. (D) Administration of 20 mg (Kasichayanula et al., 2011c).

### Canagliflozin

#### Verification


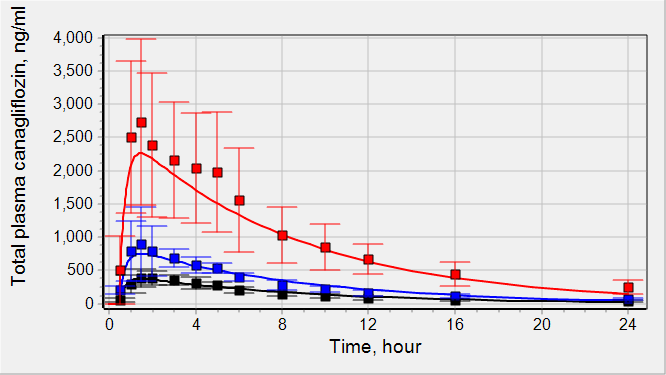


**Supplementary Figure 5 | Canagliflozin model verification against plasma data**

Total plasma canagliflozin after single administration of different doses (Devineni et al., 2013) was simulated. Colors of points correspond to the different doses: black –50 mg; blue – 100 mg; red – 300 mg.


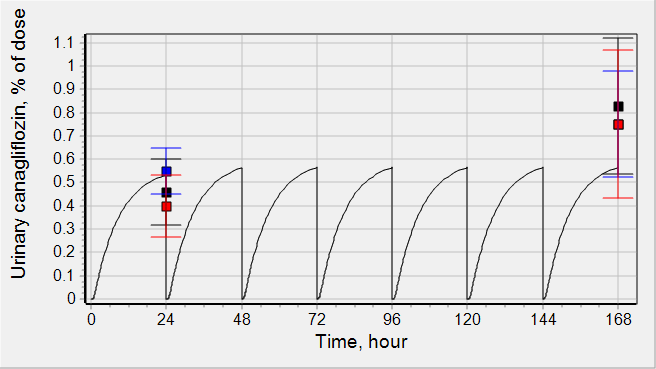


**Supplementary Figure 6 | Canagliflozin model verification against urine data**

Amount of canagliflozin recovered in urine every 24 hours after multiple administrations of different doses (Devineni et al., 2013) was simulated. Colors correspond to the different doses: black – 50 mg; blue – 100 mg; red – 300 mg.

#### Validation


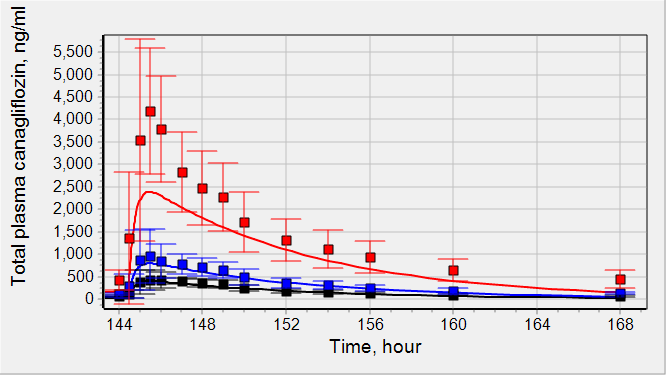

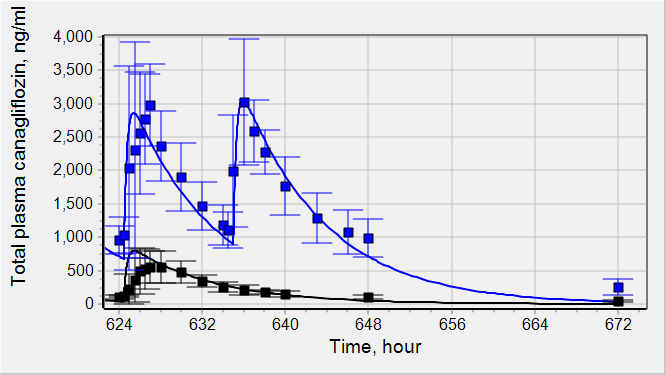


A

B

**Supplementary Figure 7 | Canagliflozin model validation**

Total plasma canagliflozin after multiple administrations of different doses was simulated. (A) Total plasma canagliflozin on 7^th^ day after multiple administrations of different doses (Devineni et al., 2013). Colors of points correspond to the different doses: black –50 mg QD; blue – 100 mg QD; red – 300 mg QD. (B) Total plasma canagliflozin on 27^th^ day and washout after multiple administrations of different doses (Devineni et al., 2012). Colors of points correspond to the different doses: black –100 mg QD; blue – 300 mg BID.

### Empagliflozin

#### Verification


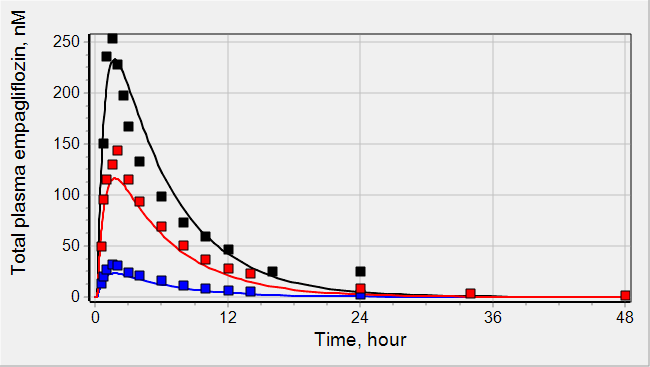

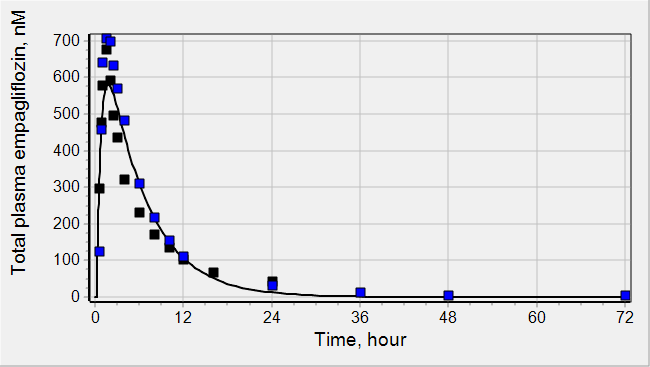


B

A


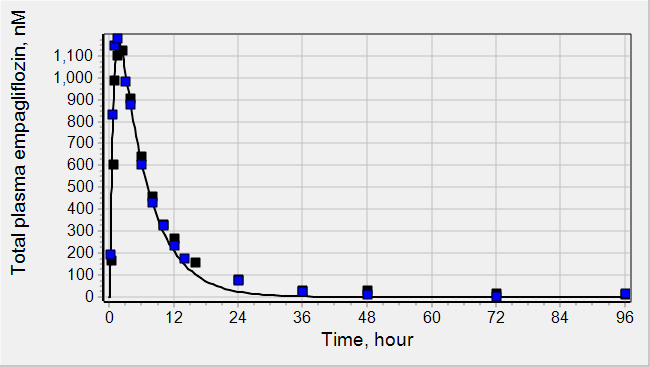

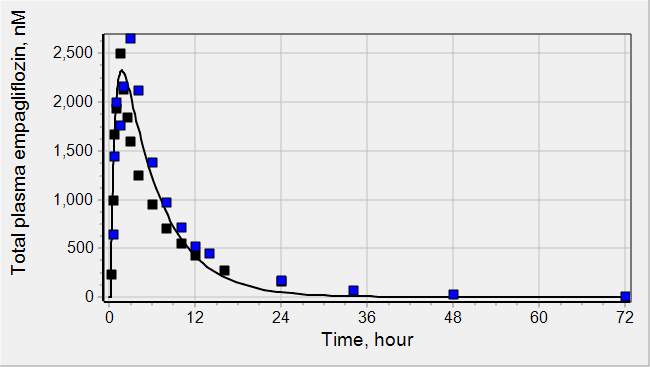


D

C

**Supplementary Figure 8 | Empagliflozin model verification against plasma data**

Total plasma empagliflozin after single administration of different doses was simulated. (A) Administration of low doses. Colors of points correspond to the different doses: black – 1 mg (Sarashina et al., 2013); blue – 5 mg (Sarashina et al., 2013); red – 10 mg (Heise et al., 2013a). (B) Administration of 25 mg. Colors of points correspond to the different sources: black – Heise et al., 2013a; blue –Sarashina et al., 2013. (C) Administration of 50 mg. Colors of points correspond to the different sources: black – Macha et al., 2014a; blue – Macha et al., 2014b. (D) Administration of 100 mg. Colors of points correspond to the different sources: black – Heise et al., 2013a; blue –Sarashina et al., 2013.


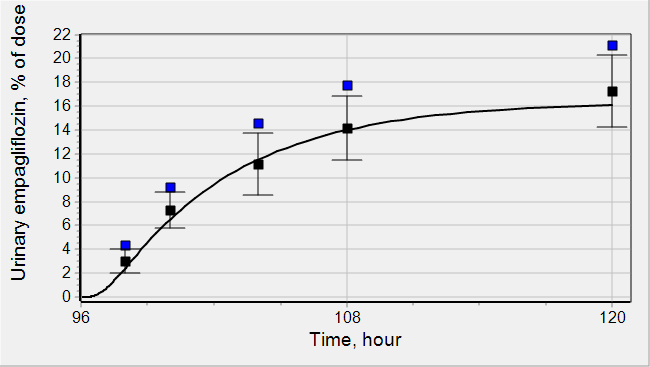

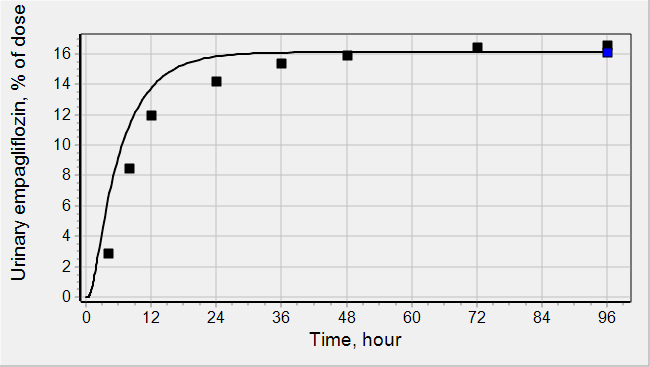


B

A


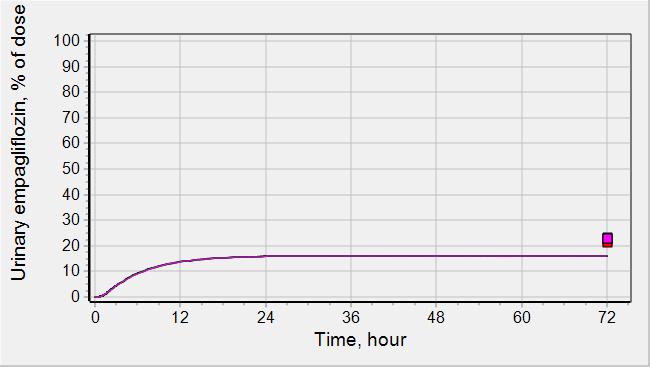


C

**Supplementary Figure 9 | Empagliflozin model verification against urine data**

Amount of empagliflozin recovered in urine after different doses was simulated. (A) Cumulative amount of empagliflozin recovered in urine during 24 hours on 5^th^ day after multiple administration of 50 mg. Colors of points correspond to the different sources: black – Brand et al., 2012; blue – Friedrich et al., 2013. (B) Cumulative amount of empagliflozin recovered in urine during 96 hours after single administration of 50 mg. Colors of points correspond to the different sources: black – Macha et al., 2014a; blue – Macha et al., 2014b. (C) Cumulative amount of empagliflozin recovered in urine during 72 hours after single administration of different doses (Sarashina et al., 2013). Colors of points correspond to the different doses (points coincide with each other): black – 1 mg, blue – 5 mg, red – 10 mg, green – 25 mg, pink – 100 mg.

#### Validation


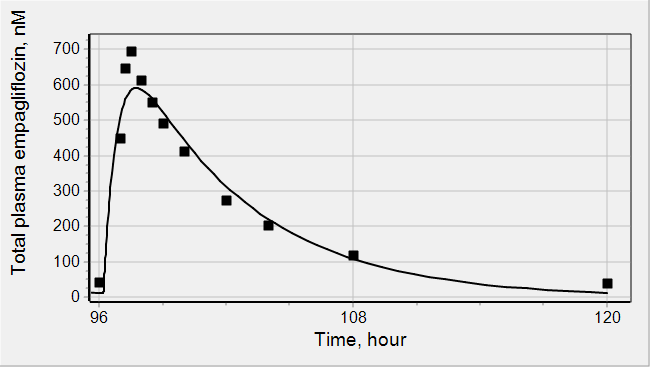

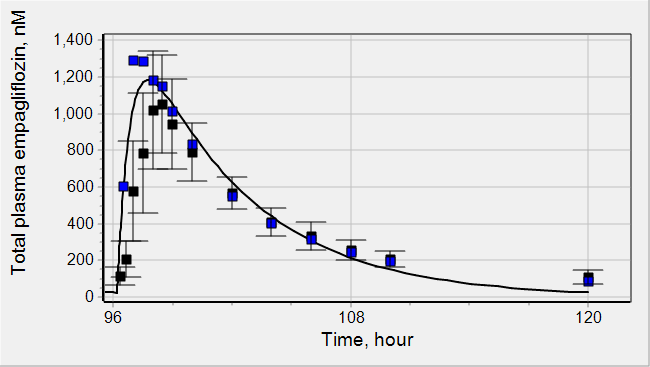


B

A


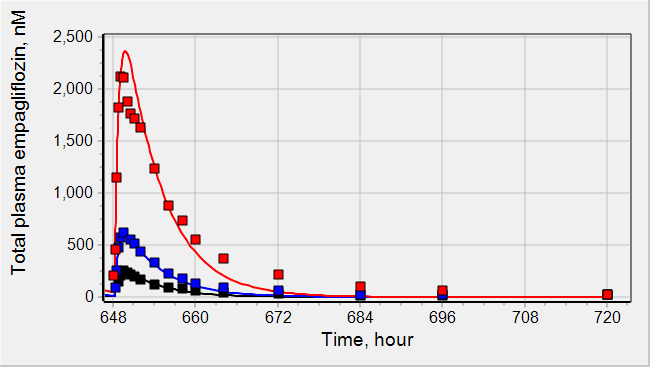

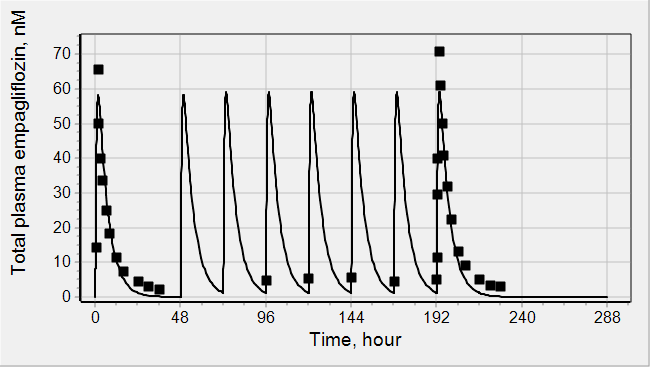


C

D


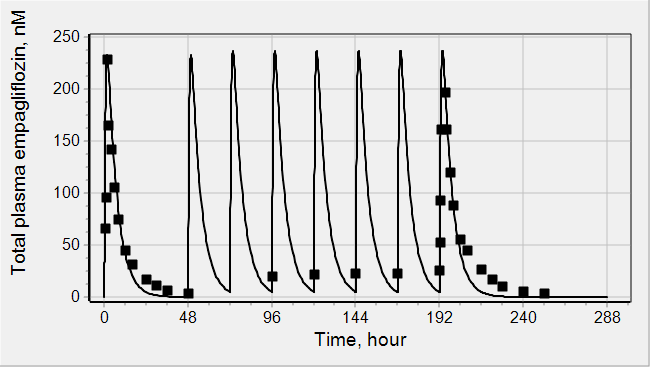

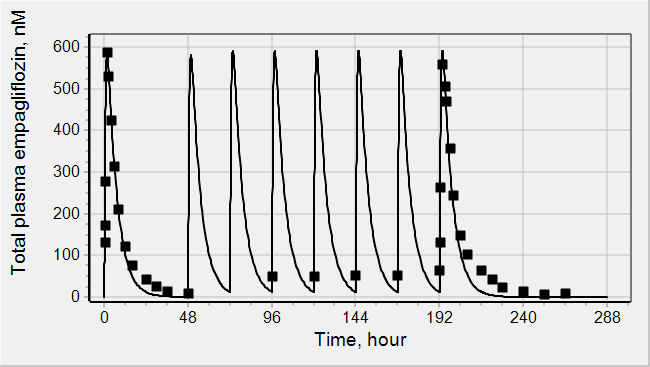


E

F


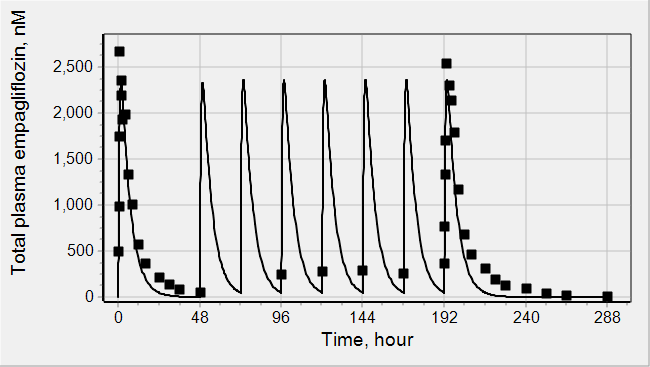


G

**Supplementary Figure 10 | Empagliflozin model validation against plasma data**

Total plasma empagliflozin after multiple administrations of different doses was simulated. (A) Total plasma empagliflozin on 5^th^ day after multiple administrations of 25 mg QD (Macha et al., 2013). (B) Total plasma empagliflozin on 5^th^ day after multiple administrations of 50 mg QD. Colors of points correspond to the different sources: black – Brand et al., 2012; blue – Friedrich et al., 2013. (C) Total plasma empagliflozin on last (28^th^) day + washout after multiple administrations of different doses (Heise et al., 2013a). Colors of points correspond to the different doses: black – 10 mg QD, blue – 25 mg QD, red – 100 mg QD. (D) Total plasma empagliflozin after multiple administrations of 2.5 mg QD (Heise et al., 2013b). (E) Total plasma empagliflozin after multiple administrations of 10 mg QD (Heise et al., 2013b). (F) Total plasma empagliflozin after multiple administrations of 25 mg QD (Heise et al., 2013b). (G) Total plasma empagliflozin after multiple administrations of 100 mg QD (Heise et al., 2013b).


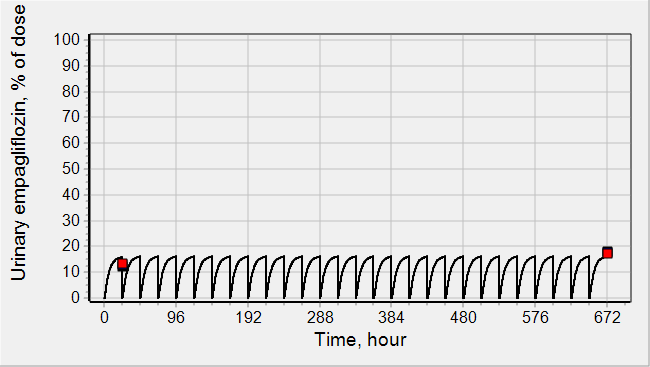

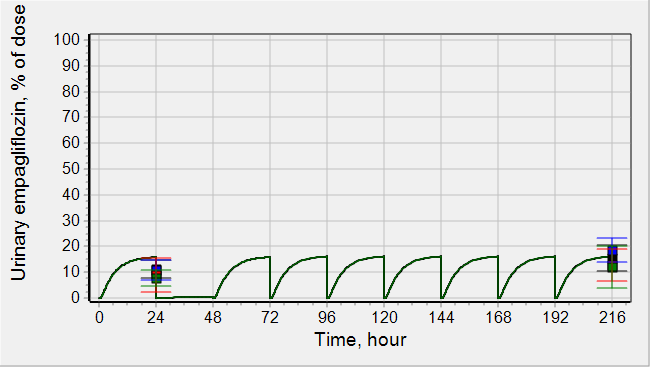


B

A

**Supplementary Figure 11 | Empagliflozin model validation against urine data**

Amount of empagliflozin recovered in urine every 24 hours after multiple administrations of different doses was simulated. (A) Heise et al., 2013a. Colors of points correspond to the different doses: black – 10 mg QD, blue – 25 mg QD, red – 100 mg QD. (B) Heise et al., 2013b. Colors of points correspond to the different doses: black – 2.5 mg QD, blue – 10 mg QD, red – 25 mg QD, green – 100 mg QD.

### Ipragliflozin

#### Verification


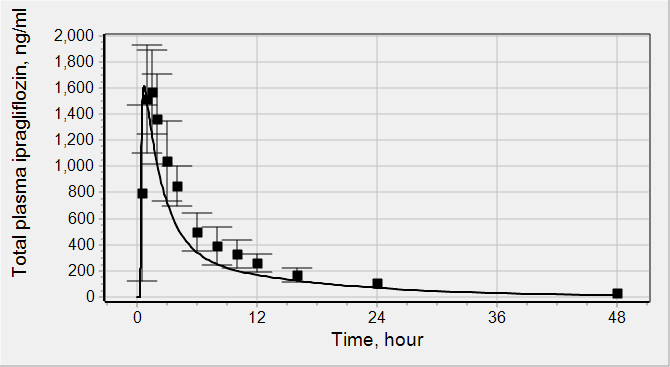

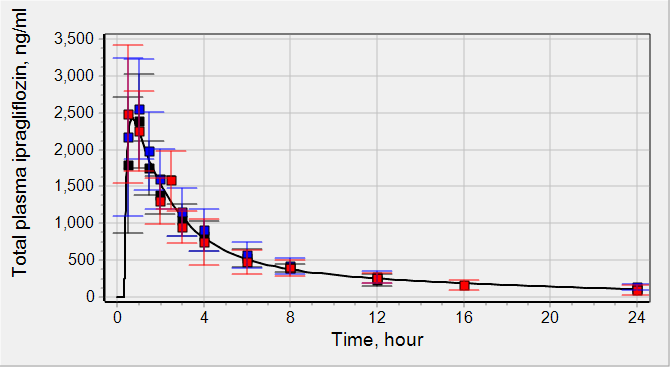


B

A

**Supplementary Figure 12 | Ipragliflozin model verification against plasma data**

Total plasma ipragliflozin after single administration of different doses was simulated. (A) Administration of 100 mg (Zhang et al., 2013). (B) Administration of 150 mg (Smulders et al., 2012). Colors of points correspond to the different studies.


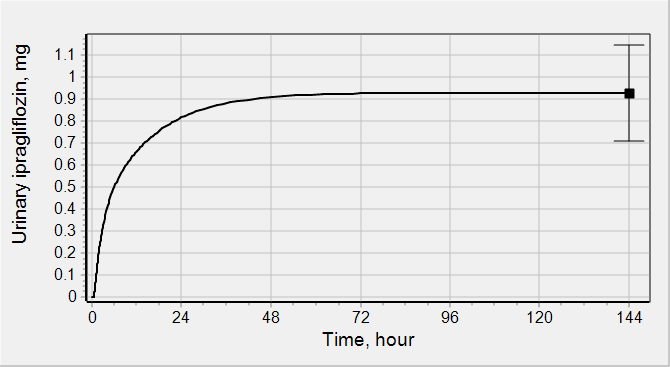


**Supplementary Figure 13 | Ipragliflozin model verification against urine data**

Cumulative amount of empagliflozin recovered in urine during 144 hours after single administration of 100 mg (Zhang et al., 2013) was simulated.

#### Validation


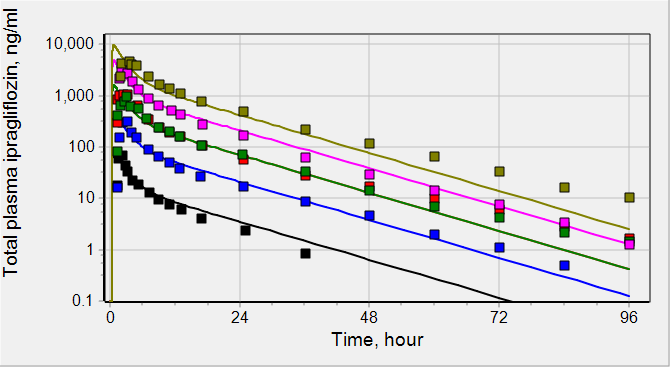

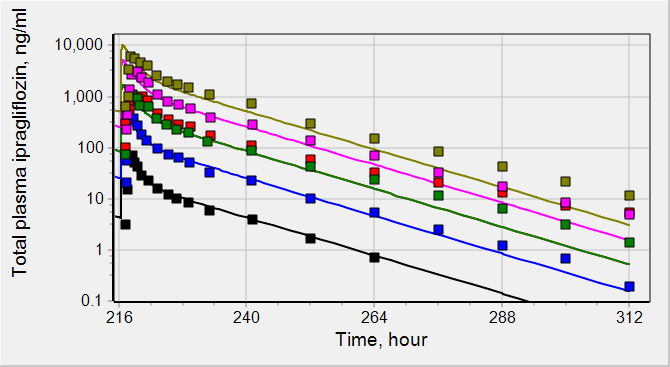


B

A

**Supplementary Figure 14 | Ipragliflozin model validation against plasma data**

Total plasma ipragliflozin after single (A) and multiple (B) administrations of different doses (Veltkamp et al., 2011) was simulated. Colors of points correspond to the different doses: black – 5 mg, blue – 30 mg, red and green – 100 mg, pink – 300 mg, brown – 600 mg.

### Tofogliflozin

#### Verification


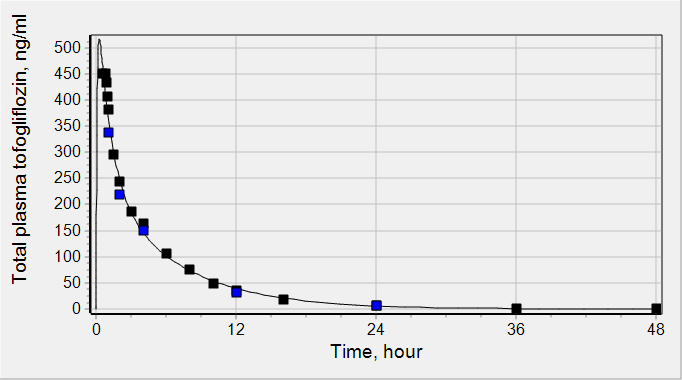


**Supplementary Figure 15 | Tofogliflozin model verification**

Total plasma tofogliflozin after single administration of 20 mg was simulated. Colors of points correspond to the different sources. Black – Schwab et al., 2013; blue – Zell et al., 2013.


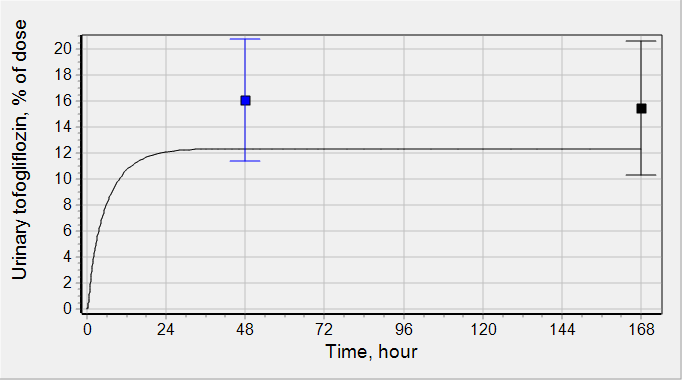


**Supplementary Figure 16 | Tofogliflozin model verification**

Amount of tofogliflozin recovered in urine during 48 and 168 hours after single administration of 20 mg was simulated. Colors of points correspond to the different sources. Black – Schwab et al., 2013; blue – Zell et al., 2013.

### Simulations


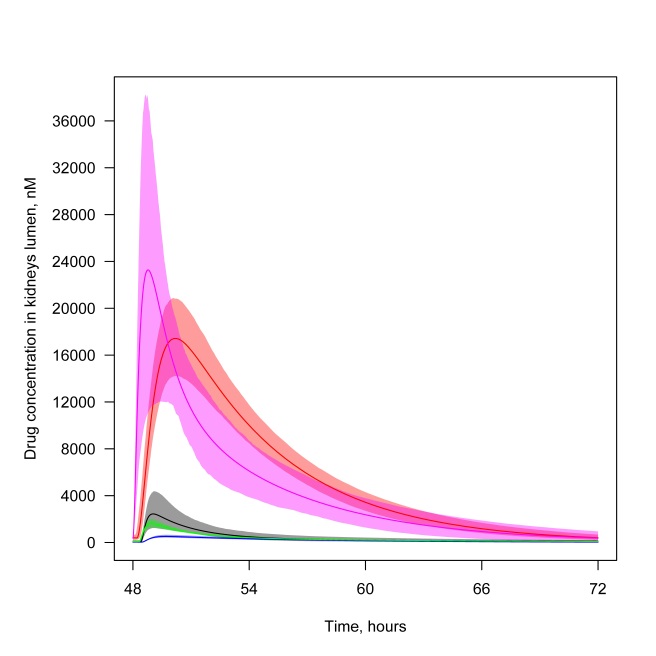


**Supplementary Figure 17 | SGLT2 inhibitors concentrations in kidneys proximal tubules lumen**

SGLT2 inhibitors levels in kidneys proximal tubules lumen after multiple administrations of 20 mg QD were simulated. Colors of curves correspond to different compounds: black – dapagliflozin, blue – canagliflozin, red – empagliflozin, green – ipragliflozin, pink - tofogliflozin.


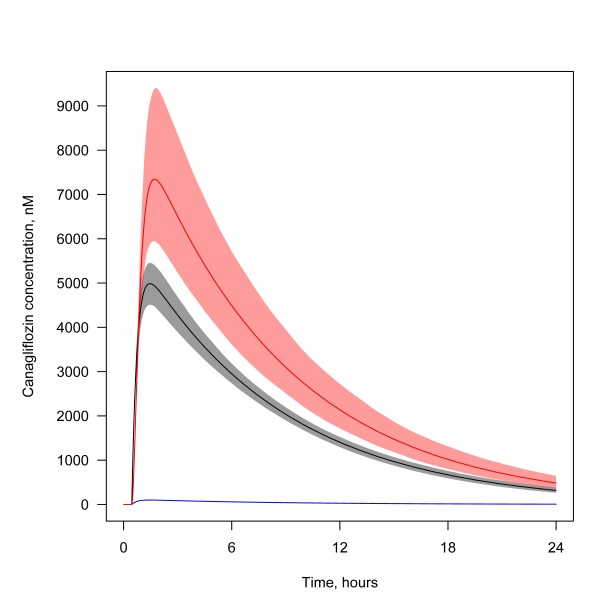


**Supplementary Figure 18 | Canagliflozin levels in plasma and kidneys proximal tubules lumen**

Canagliflozin levels in different compartments after single administration of 300 mg were simulated. Colors of curves correspond to various compartments: black – total plasma concentration, blue – unbound plasma concentration, red – concentration in kidneys proximal tubules lumen.


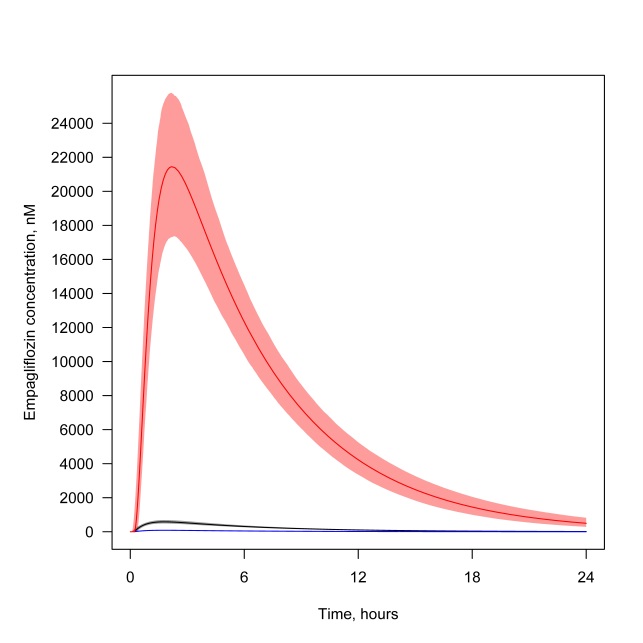


**Supplementary Figure 19 | Empagliflozin levels in plasma and kidneys proximal tubules lumen**

Empagliflozin levels in different compartments after single administration of 25 mg were simulated. Colors of curves correspond to various compartments: black – total plasma concentration, blue – unbound plasma concentration, red – concentration in kidneys proximal tubules lumen.


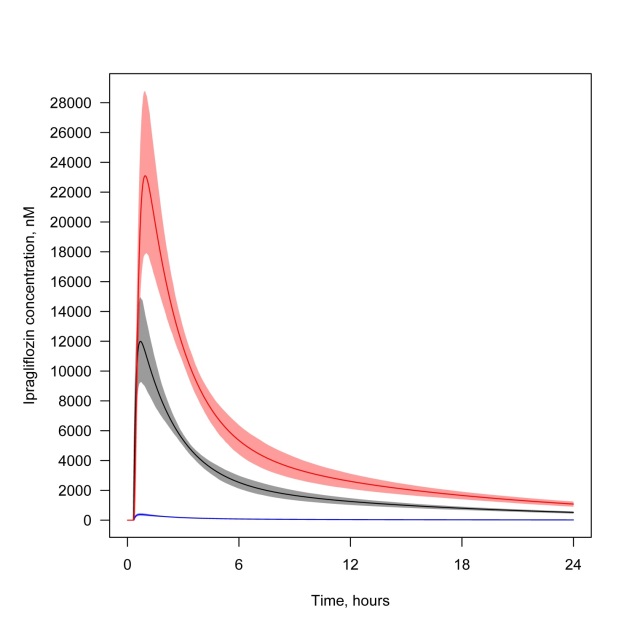


**Supplementary Figure 20 | Ipragliflozin levels in plasma and kidneys proximal tubules lumen**

Ipragliflozin levels in different compartments after single administration of 300 mg were simulated. Colors of curves correspond to various compartments: black – total plasma concentration, blue – unbound plasma concentration, red – concentration in kidneys proximal tubules lumen.


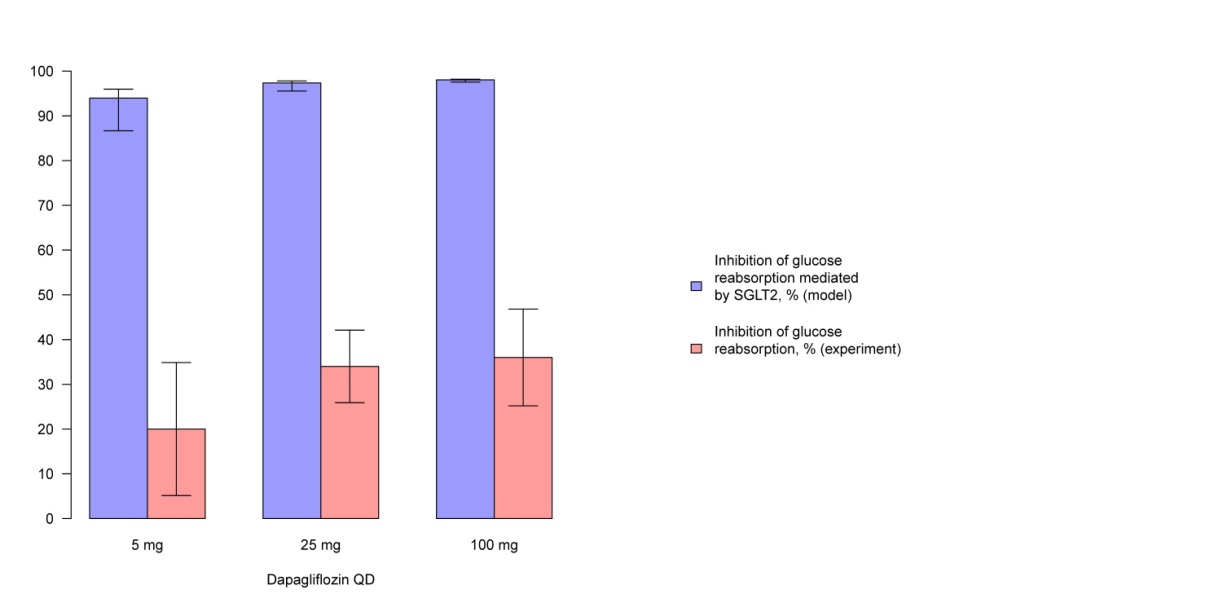


**Supplementary Figure 21 | Comparison of average inhibition of glucose reabsorption mediated by SGLT2 and glucose reabsorption inhibition levels during treatment with dapagliflozin**

Average inhibition of glucose reabsorption mediated by SGLT2 (simulated by the model) and glucose reabsorption inhibition (measured in experiment) levels on 1^st^ day after multiple administrations of different doses of dapagliflozin (Komoroski et al., 2009b) were compared.


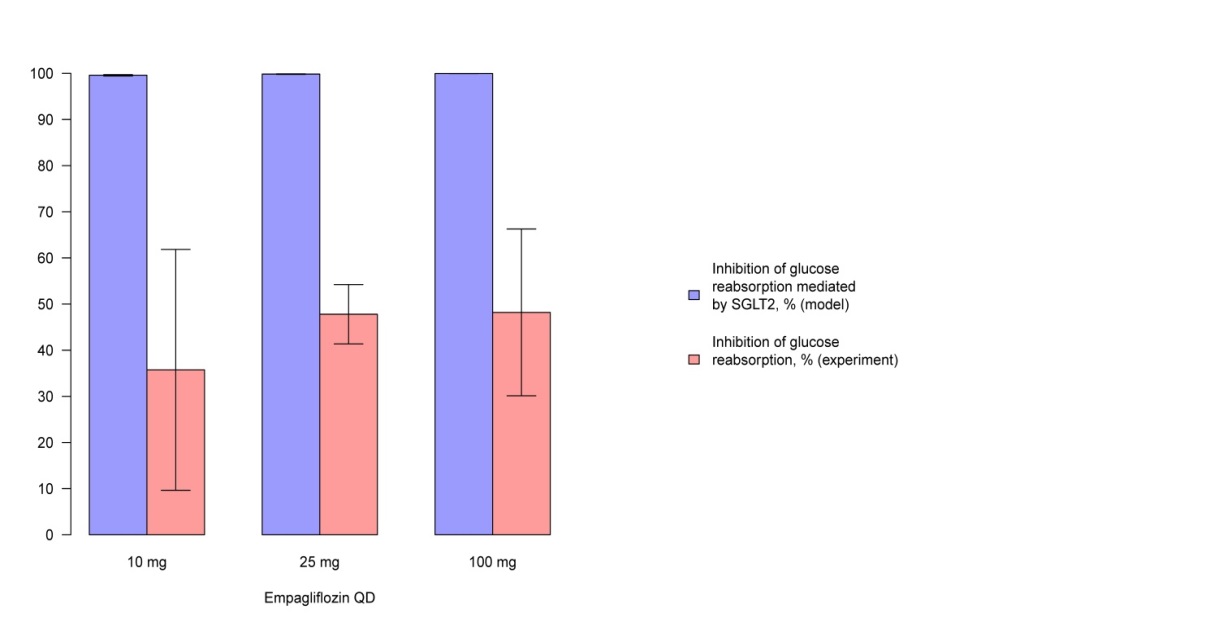


A


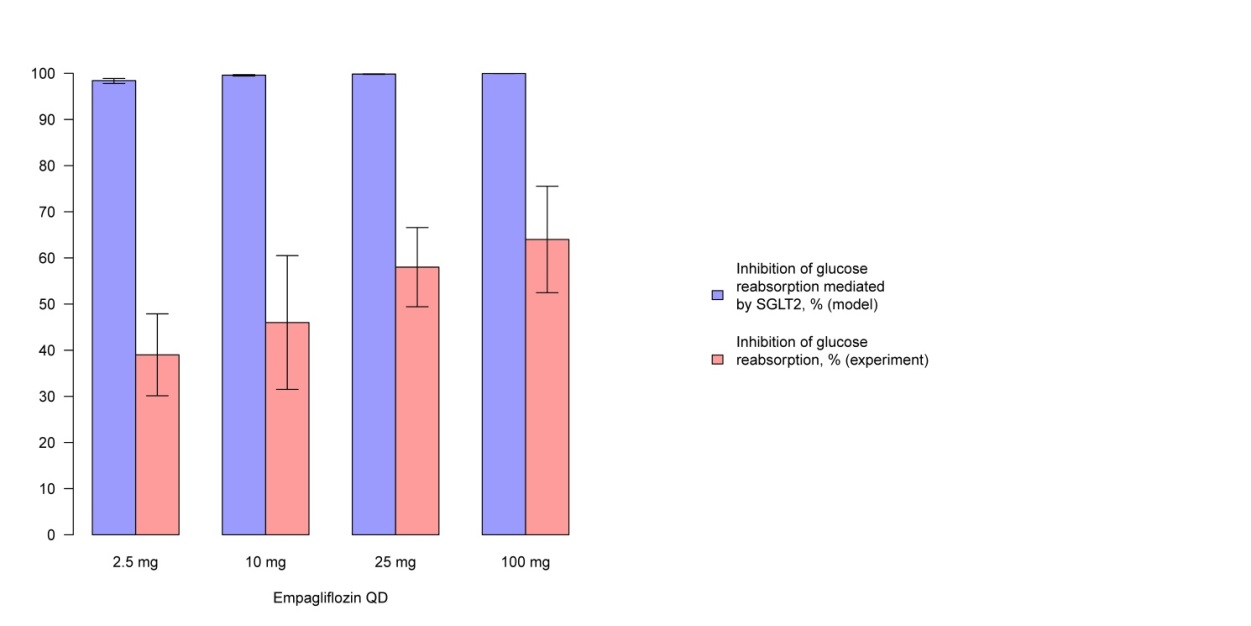


B

**Supplementary Figure 22 | Comparison of average inhibition of glucose reabsorption mediated by SGLT2 and glucose reabsorption inhibition levels during treatment with empagliflozin**

Average inhibition of glucose reabsorption mediated by SGLT2 (simulated by the model) and glucose reabsorption inhibition (measured in experiment) levels during treatment with empagliflozin. (A) On 27^th^ day (Heise et al., 2013a). (B) On 8^th^ day (Heise et al., 2013b) were compared.


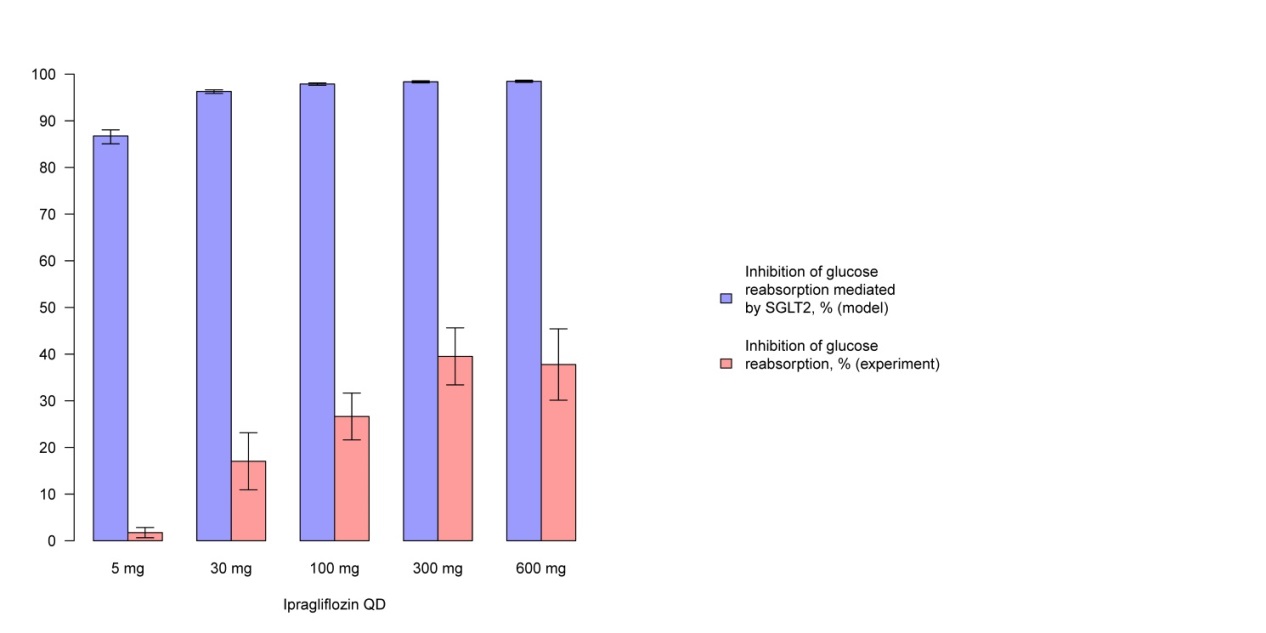


A


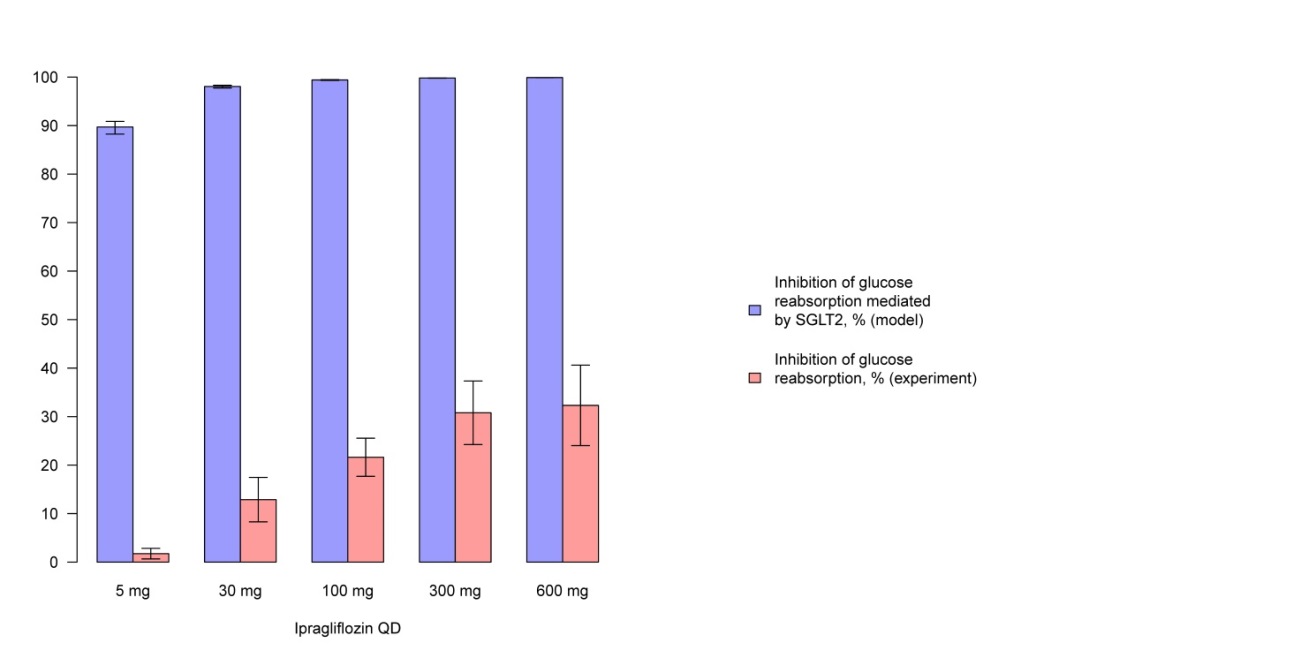


B

**Supplementary Figure 23 | Comparison of average inhibition of glucose reabsorption mediated by SGLT2 and glucose reabsorption inhibition levels during treatment with ipragliflozin**

Average inhibition of glucose reabsorption mediated by SGLT2 (simulated by the model) and glucose reabsorption inhibition (measured in experiment) levels during treatment with ipragliflozin (Veltkamp et al., 2011). (A) On 1^st^ day. (B) On 10^th^ day were compared.


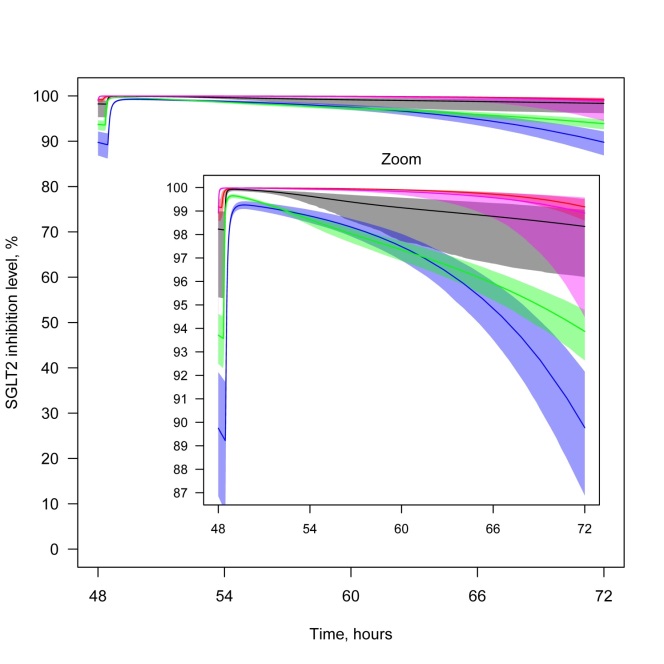


**Supplementary Figure 24 | SGLT2 inhibition level after drugs administration**

SGLT2 inhibition level after multiple administrations of 20 mg QD of compounds was simulated. Colors of curves correspond to different compounds: black – dapagliflozin, blue – canagliflozin, red – empagliflozin, green – ipragliflozin, pink - tofogliflozin.


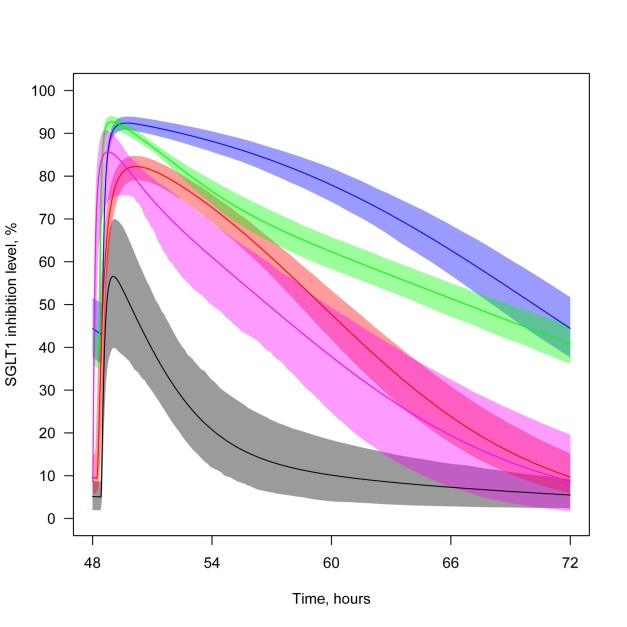


**Supplementary Figure 25 | SGLT1 inhibition level after drugs administration**

SGLT1 inhibition level on 3^rd^ day after multiple administrations of labeled doses of marketed SGLT2 inhibitors and maximal doses approved for phase 2/3 studies of other SGLT2 inhibitors was simulated. Colors of curves correspond to different compounds: black – 10 mg QD dapagliflozin, blue – 300 mg QD canagliflozin, red – 25 mg QD empagliflozin, green – 300 mg QD ipragliflozin, pink – 40 mg QD tofogliflozin.


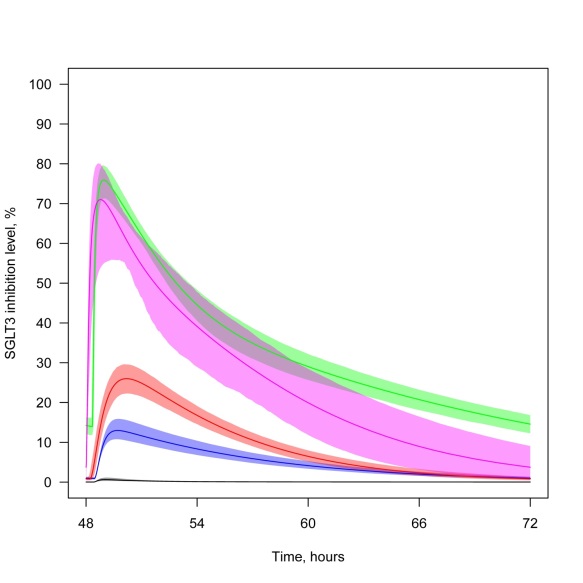


**Supplementary Figure 26 | SGLT3 inhibition level after drugs administration**

SGLT3 inhibition level after multiple administrations of labeled doses of marketed SGLT2 inhibitors and maximal doses approved for phase 2/3 studies of other SGLT2 inhibitors was simulated. Colors of curves correspond to different compounds: black – 10 mg QD dapagliflozin, blue – 300 mg QD canagliflozin, red – 25 mg QD empagliflozin, green – 300 mg QD ipragliflozin, pink – 40 mg QD tofogliflozin.


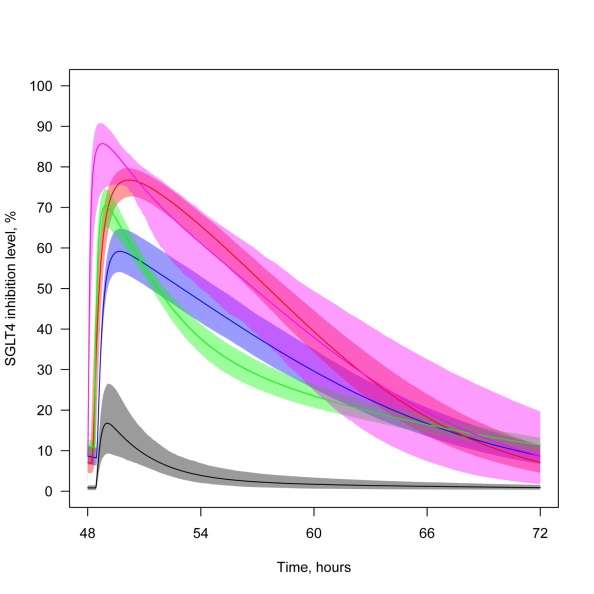


**Supplementary Figure 27 | SGLT4 inhibition level after drugs administration**

SGLT4 inhibition level after multiple administrations of labeled doses of marketed SGLT2 inhibitors and maximal doses approved for phase 2/3 studies of other SGLT2 inhibitors was simulated. Colors of curves correspond to different compounds: black – 10 mg QD dapagliflozin, blue – 300 mg QD canagliflozin, red – 25 mg QD empagliflozin, green – 300 mg QD ipragliflozin, pink – 40 mg QD tofogliflozin.


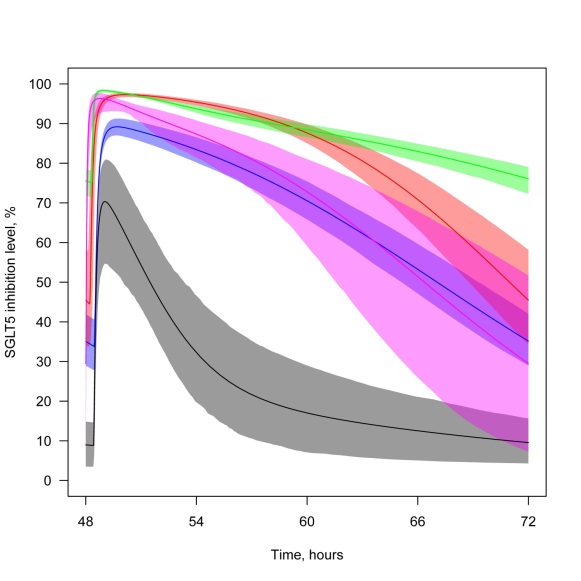


**Supplementary Figure 28 | SGLT5 inhibition level after drugs administration**

SGLT5 inhibition level after multiple administrations of labeled doses of marketed SGLT2 inhibitors and maximal doses approved for phase 2/3 studies of other SGLT2 inhibitors was simulated. Colors of curves correspond to different compounds: black – 10 mg QD dapagliflozin, blue – 300 mg QD canagliflozin, red – 25 mg QD empagliflozin, green – 300 mg QD ipragliflozin, pink – 40 mg QD tofogliflozin.


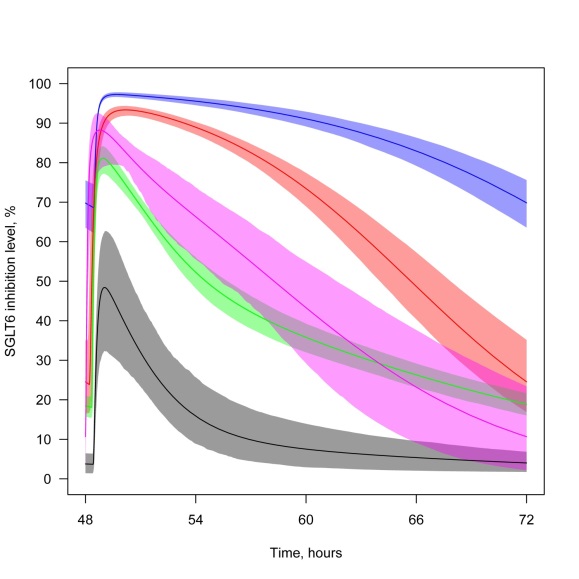


**Supplementary Figure 29 | SGLT6 inhibition level after drugs administration**

SGLT6 inhibition level after multiple administrations of labeled doses of marketed SGLT2 inhibitors and maximal doses approved for phase 2/3 studies of other SGLT2 inhibitors was simulated. Colors of curves correspond to different compounds: black – 10 mg QD dapagliflozin, blue – 300 mg QD canagliflozin, red – 25 mg QD empagliflozin, green – 300 mg QD ipragliflozin, pink – 40 mg QD tofogliflozin.

# References

Beresford, W.A. (2000). *Histology.* Morgantown: West Virginia University.

Borghi, L., Meschi, T., Amato, F., Briganti, A., Novarini, A., Giannini, A. (1996). Urinary volume, water and recurrences in idiopathic calcium nephrolithiasis: a 5-year randomized prospective study. *J Urol*. 155, 839-43.

Boulton, D.W., Kasichayanula, S., Keung, C.F., Arnold, M.E., Christopher, L.J., Xu, X.S., Lacreta, F. (2013). Simultaneous oral therapeutic and intravenous №4C-microdoses to determine the absolute oral bioavailability of saxagliptin and dapagliflozin. *Br J Clin Pharmacol*. 75, 763-768. doi: 10.1111/j.1365-2125.2012.04391.x.

Brand, T., Macha, S., Mattheus, M., Pinnetti, S., Woerle, H.J. (2012). Pharmacokinetics of empagliflozin, a sodium glucose cotransporter-2 (SGLT-2) inhibitor, coadministered with sitagliptin in healthy volunteers. *Adv Ther*. 29, 889-899. doi: 10.1007/s12325-012-0055-3.

Devineni, D., Curtin, C.R., Polidori, D., Gutierrez, M.J., Murphy, J., Rusch, S., Rothenberg, P.L. (2013). Pharmacokinetics and pharmacodynamics of canagliflozin, a sodium glucose co-transporter 2 inhibitor, in subjects with type 2 diabetes mellitus. *J Clin Pharmacol*. 53, 601-610. doi: 10.1002/jcph.88.

Devineni, D., Morrow, L., Hompesch, M., Skee, D., Vandebosch, A., Murphy, J., Ways, K., Schwartz, S. (2012). Canagliflozin improves glycaemic control over 28 days in subjects with type 2 diabetes not optimally controlled on insulin. *Diabetes Obes Metab*. 14, 539-545. doi: 10.1111/j.1463-1326.2012.01558.x.

Friedrich, C., Metzmann, K., Rose, P., Mattheus, M., Pinnetti, S., Woerle, H.J. (2013). A randomized, open-label, crossover study to evaluate the pharmacokinetics of empagliflozin and linagliptin after coadministration in healthy male volunteers. *Clin Ther*. 35, A33-A42. doi: 10.1016/j.clinthera.2012.12.002.

Goodwin, N.C., Mabon, R., Harrison, B.A., Shadoan, M.K., Almstead, Z.Y., Xie, Y., Healy, J., Buhring, L.M., DaCosta, C.M., Bardenhagen, J., Mseeh, F., Liu, Q., Nouraldeen, A., Wilson, A.G., Kimball, S.D., Powell, D.R., Rawlins, D.B. (2009). Novel L-xylose derivatives as selective sodium-dependent glucose cotransporter 2 (SGLT2) inhibitors for the treatment of type 2 diabetes. *J Med Chem*. 52, 6201-6204. doi: 10.1021/jm900951n.

Grempler, R., Thomas, L., Eckhardt, M., Himmelsbach, F., Sauer, A., Sharp, D.E., Bakker, R.A., Mark, M., Klein, T., Eickelmann, P. (2012). Empagliflozin, a novel selective sodium glucose cotransporter-2 (SGLT-2) inhibitor: characterisation and comparison with other SGLT-2 inhibitors. *Diabetes Obes Metab*. 14, 83-90. doi: 10.1111/j.1463-1326.2011.01517.x.

Heise, T., Seewaldt-Becker, E., Macha, S., Hantel, S., Pinnetti, S., Seman, L., Woerle, H.J. (2013a). Safety, tolerability, pharmacokinetics and pharmacodynamics following 4 weeks' treatment with empagliflozin once daily in patients with type 2 diabetes. *Diabetes Obes Metab*. 15, 613-621. doi: 10.1111/dom.12073.

Heise, T., Seman, L., Macha, S., Jones, P., Marquart, A., Pinnetti, S., Woerle, H.J., Dugi, K. (2013b). Safety, Tolerability, Pharmacokinetics, and Pharmacodynamics of Multiple Rising Doses of Empagliflozin in Patients with Type 2 Diabetes Mellitus. *Diabetes Ther*. 4, 331-345. doi: 10.1007/s13300-013-0030-2.

Hughson, M., Farris, A.B. 3rd, Douglas-Denton, R., Hoy, W.E., Bertram, J.F. (2003). Glomerular number and size in autopsy kidneys: the relationship to birth weight. *Kidney Int*. 63, 2113-2122.

Imamura, A., Kusunoki, M., Ueda, S., Hayashi, N., Imai, Y. (2013). Impact of voglibose on the pharmacokinetics of dapagliflozin in Japanese patients with type 2 diabetes. *Diabetes Ther*. 4, 41-49. doi: 10.1007/s13300-012-0016-5.

Imamura, M., Nakanishi, K., Suzuki, T., Ikegai, K., Shiraki, R., Ogiyama, T., Murakami, T., Kurosaki, E., Noda, A., Kobayashi, Y., Yokota, M., Koide, T., Kosakai, K., Ohkura, Y., Takeuchi, M., Tomiyama, H., Ohta, M. (2012). Discovery of Ipragliflozin (ASP1941): a novel C-glucoside with benzothiophene structure as a potent and selective sodium glucose co-transporter 2 (SGLT2) inhibitor for the treatment of type 2 diabetes mellitus. Bioorg Med Chem. 20, 3263-3279. doi: 10.1016/j.bmc.2012.03.051.

Kasichayanula, S., Liu, X., Zhang, W., Pfister, M., LaCreta, F.P., Boulton, D.W. (2011a). Influence of hepatic impairment on the pharmacokinetics and safety profile of dapagliflozin: an open-label, parallel-group, single-dose study. *Clin Ther*. 33, 1798-808. doi: 10.1016/j.clinthera.2011.09.011.

Kasichayanula, S., Liu, X., Griffen, S.C., Lacreta, F.P., Boulton, D.W. (2013a). Effects of rifampin and mefenamic acid on the pharmacokinetics and pharmacodynamics of dapagliflozin. *Diabetes Obes Metab*. 15, 280-283. doi: 10.1111/dom.12024.

Kasichayanula, S., Liu, X., Shyu, W.C., Zhang, W., Pfister, M., Griffen, S.C., Li, T., LaCreta, F.P., Boulton, D.W. (2011b). Lack of pharmacokinetic interaction between dapagliflozin, a novel sodium-glucose transporter 2 inhibitor, and metformin, pioglitazone, glimepiride or sitagliptin in healthy subjects. *Diabetes Obes Metab*. 13, 47-54. doi: 10.1111/j.1463-1326.2010.01314.x.

Kasichayanula, S., Chang, M., Hasegawa, M., Liu, X., Yamahira, N., LaCreta, F.P., Imai, Y., Boulton, D.W. (2011c). Pharmacokinetics and pharmacodynamics of dapagliflozin, a novel selective inhibitor of sodium-glucose co-transporter type 2, in Japanese subjects without and with type 2 diabetes mellitus. *Diabetes Obes Metab*. 13, 357-365. doi: 10.1111/j.1463-1326.2011.01359.x.

Kasichayanula, S., Chang, M., Liu, X., Shyu, W.C., Griffen, S.C., LaCreta, F.P., Boulton, D.W. (2012). Lack of pharmacokinetic interactions between dapagliflozin and simvastatin, valsartan, warfarin, or digoxin. *Adv Ther*. 29, 163-177. doi: 10.1007/s12325-011-0098-x.

Kasichayanula, S., Liu, X., Pe Benito, M., Yao, M., Pfister, M., LaCreta, F.P., Humphreys, W.G., Boulton, D.W. (2013b). The influence of kidney function on dapagliflozin exposure, metabolism and pharmacodynamics in healthy subjects and in patients with type 2 diabetes mellitus. *Br J Clin Pharmacol*. 76, 432-444. doi: 10.1111/bcp.12056.

Kasichayanula, S., Liu, X., Zhang, W., Pfister, M., Reele, S.B., Aubry, A.F., LaCreta, F.P., Boulton, D.W. (2011d). Effect of a high-fat meal on the pharmacokinetics of dapagliflozin, a selective SGLT2 inhibitor, in healthy subjects. *Diabetes Obes Metab*. 13, 770-773. doi: 10.1111/j.1463-1326.2011.01397.x.

Komoroski, B., Vachharajani, N., Boulton, D., Kornhauser, D., Geraldes, M., Li, L., Pfister, M. (2009a). Dapagliflozin, a novel SGLT2 inhibitor, induces dose-dependent glucosuria in healthy subjects. *Clin Pharmacol Ther*. 85, 520-526. doi: 10.1038/clpt.2008.251.

Komoroski, B., Vachharajani, N., Feng, Y., Li, L., Kornhauser, D., Pfister, M. (2009b). Dapagliflozin, a novel, selective SGLT2 inhibitor, improved glycemic control over 2 weeks in patients with type 2 diabetes mellitus. *Clin Pharmacol Ther*. 85, 513-519. doi: 10.1038/clpt.2008.250.

Kurepina, M.M. (2003). *Human anatomy*. Moscow: Vlados.

Macha, S., Rose, P., Mattheus, M., Cinca, R., Pinnetti, S., Broedl, U.C., Woerle, H.J. (2014a). Pharmacokinetics, safety and tolerability of empagliflozin, a sodium glucose cotransporter 2 inhibitor, in patients with hepatic impairment. *Diabetes Obes Metab*. 16,118-123. doi: 10.1111/dom.12183.

Macha, S., Mattheus, M., Halabi, A., Pinnetti, S., Woerle, H.J., Broedl, U.C. (2014b). Pharmacokinetics, pharmacodynamics and safety of empagliflozin, a sodium glucose cotransporter 2 (SGLT2) inhibitor, in subjects with renal impairment. *Diabetes Obes Metab*. 16, 215-222. doi: 10.1111/dom.12182.

Macha, S., Rose, P., Mattheus, M., Pinnetti, S., Woerle, H.J. (2013). Lack of drug-drug interaction between empagliflozin, a sodium glucose cotransporter 2 inhibitor, and warfarin in healthy volunteers. *Diabetes Obes Metab*. 15, 316-323. doi: 10.1111/dom.12028.

Meng, W., Ellsworth, B.A., Nirschl, A.A., McCann, P.J., Patel, M., Girotra, R.N., Wu, G., Sher, P.M., Morrison, E.P., Biller, S.A., Zahler, R., Deshpande, P.P., Pullockaran, A., Hagan, D.L., Morgan, N., Taylor, J.R., Obermeier, M.T., Humphreys, W.G., Khanna, A., Discenza, L., Robertson, J.G., Wang, A., Han, S., Wetterau, J.R., Janovitz, E.B., Flint, O.P., Whaley, J.M., Washburn, W.N. (2008). Discovery of dapagliflozin: a potent, selective renal sodium-dependent glucose cotransporter 2 (SGLT2) inhibitor for the treatment of type 2 diabetes. *J Med Chem*. 51,1145-1149. doi: 10.1021/jm701272q.

Nomura S, Sakamaki S, Hongu M, Kawanishi E, Koga Y, Sakamoto T, Yamamoto Y, Ueta K, Kimata H, Nakayama K, Tsuda-Tsukimoto M. (2010). Discovery of canagliflozin, a novel C-glucoside with thiophene ring, as sodium-dependent glucose cotransporter 2 inhibitor for the treatment of type 2 diabetes mellitus. *J Med Chem*. 53, 6355-6360. doi: 10.1021/jm100332n.

Obermeier, M., Yao, M., Khanna, A., Koplowitz, B., Zhu, M., Li, W., Komoroski, B., Kasichayanula, S., Discenza, L., Washburn, W., Meng, W., Ellsworth, B.A., Whaley, J.M., Humphreys, W.G. (2010). In vitro characterization and pharmacokinetics of dapagliflozin (BMS-512148), a potent sodium-glucose cotransporter type II inhibitor, in animals and humans. *Drug Metab Dispos*. 38, 405-414. doi: 10.1124/dmd.109.029165.

Ohtake, Y., Sato, T., Kobayashi, T., Nishimoto, M., Taka, N., Takano, K., Yamamoto, K., Ohmori, M., Yamaguchi, M., Takami, K., Yeu, S.Y., Ahn, K.H., Matsuoka, H., Morikawa, K., Suzuki, M., Hagita, H., Ozawa, K., Yamaguchi, K., Kato, M., Ikeda, S. (2012). Discovery of tofogliflozin, a novel C-arylglucoside with an O-spiroketal ring system, as a highly selective sodium glucose cotransporter 2 (SGLT2) inhibitor for the treatment of type 2 diabetes. *J Med Chem*. 55, 7828-7840. doi: 10.1021/jm300884k.

Retzlaff, J.A., Tauxe, W.N., Kiely, J.M., Stroebel, C.F. (1969). Erythrocyte volume, plasma volume, and lean body mass in adult men and women. *Blood*. 33, 649-661.

Sarashina, A., Koiwai, K., Seman, L.J., Yamamura, N., Taniguchi, A., Negishi, T., Sesoko, S., Woerle, H.J., Dugi, K.A. (2013). Safety, tolerability, pharmacokinetics and pharmacodynamics of single doses of empagliflozin, a sodium glucose cotransporter 2 (SGLT2) inhibitor, in healthy Japanese subjects. *Drug Metab Pharmacokinet*. 28, 213-219.

Schwab, D., Portron, A., Backholer, Z., Lausecker, B., Kawashima, K. (2013). A novel double-tracer technique to characterize absorption, distribution, metabolism and excretion (ADME) of [14C]tofogliflozin after oral administration and concomitant intravenous microdose administration of [13C]tofogliflozin in humans. *Clin Pharmacokinet*. 52, 463-473. doi: 10.1007/s40262-013-0051-z.

Smulders, R.A., Zhang, W., Veltkamp, S.A., van Dijk, J., Krauwinkel, W.J., Keirns, J., Kadokura, T. (2012). No pharmacokinetic interaction between ipragliflozin and sitagliptin, pioglitazone, or glimepiride in healthy subjects. *Diabetes Obes Metab*. 14, 937-943. doi: 10.1111/j.1463-1326.2012.01624.x.

Suzuki, M., Honda, K., Fukazawa, M., Ozawa, K., Hagita, H., Kawai, T., Takeda, M., Yata, T., Kawai, M., Fukuzawa, T., Kobayashi, T., Sato, T., Kawabe, Y., Ikeda, S. (2012). Tofogliflozin, a potent and highly specific sodium/glucose cotransporter 2 inhibitor, improves glycemic control in diabetic rats and mice. *J Pharmacol Exp Ther*. 341, 692-701. doi: 10.1124/jpet.112.191593.

Tahara, A., Kurosaki, E., Yokono, M., Yamajuku, D., Kihara, R., Hayashizaki, Y., Takasu, T., Imamura, M., Qun, L., Tomiyama, H., Kobayashi, Y., Noda, A., Sasamata, M., Shibasaki, M. (2012). Pharmacological profile of ipragliflozin (ASP1941), a novel selective SGLT2 inhibitor, in vitro and in vivo. *Naunyn Schmiedebergs Arch Pharmacol*. 385, 423-436. doi: 10.1007/s00210-011-0713-z.

Veltkamp, S.A., Kadokura, T., Krauwinkel, W.J., Smulders, R.A. (2011). Effect of Ipragliflozin (ASP1941), a novel selective sodium-dependent glucose co-transporter 2 inhibitor, on urinary glucose excretion in healthy subjects. *Clin Drug Investig*. 31, 839-851. doi: 10.2165/11594330-000000000-00000.

Yang, L., Li, H., Li, H., Bui, A., Chang, M., Liu, X., Kasichayanula, S., Griffen, S.C., LaCreta, F.P., Boulton, D.W. (2013). Pharmacokinetic and pharmacodynamic properties of single- and multiple-dose of dapagliflozin, a selective inhibitor of SGLT2, in healthy Chinese subjects. *Clin Ther*. 35, 1211-1222.e2. doi: 10.1016/j.clinthera.2013.06.017.

Zell, M., Husser, C., Kuhlmann, O., Schwab, D., Uchimura, T., Kemei, T., Kawashima, K., Yamane, M., Pahler, A. (2013). Metabolism and mass balance of SGLT2 inhibitor tofogliflozin following oral administration to humans. *Xenobiotica*. 44, 369-378. doi: 10.3109/00498254.2013.839847.

Zhang, W., Krauwinkel, W.J., Keirns, J., Townsend, R.W., Lasseter, K.C., Plumb, L., Kadokura, T., Ushigome, F., Smulders, R. (2013). The effect of moderate hepatic impairment on the pharmacokinetics of ipragliflozin, a novel sodium glucose co-transporter 2 (SGLT2) inhibitor. *Clin Drug Investig*. 33, 489-496. doi: 10.1007/s40261-013-0089-6.

# Model code from DBSolve

//! Explicit functions

AUC_sglt2_inhibition_24h=AUC/24;

Drug_plasma_total_ng_ml=1000*Drug_pls;

Drug_plasma_unbound_mg_l=fup_drug*Drug_pls;

Drug_plasma_total_uM=1000*Drug_pls/Mr_drug;

Drug_plasma_total_nM=1000000*Drug_pls/Mr_drug;

Drug_plasma_unbound_uM=1000*fup_drug*Drug_pls/Mr_drug;

Drug_plasma_unbound_nM=1000000*fup_drug*Drug_pls/Mr_drug;

Drug_lumen_uM=1000*Drug_lum/Mr_drug;

Drug_lumen_nM=1000000*Drug_lum/Mr_drug;

Urinary_drug_percent_of_dose=100*drug_urine/Dose_drug;

///

SGLT1_inhibition_level_percent=100*Drug_lumen_nM/(IC50_sglt1_drug+Drug_lumen_nM);

SGLT2_inhibition_level_percent=100*Drug_lumen_nM/(IC50_sglt2_drug+Drug_lumen_nM);

SGLT3_inhibition_level_percent=100*Drug_lumen_nM/(IC50_sglt3_drug+Drug_lumen_nM);

SGLT4_inhibition_level_percent=100*Drug_lumen_nM/(IC50_sglt4_drug+Drug_lumen_nM);

SGLT5_inhibition_level_percent=100*Drug_lumen_nM/(IC50_sglt5_drug+Drug_lumen_nM);

SGLT6_inhibition_level_percent=100*Drug_lumen_nM/(IC50_sglt6_drug+Drug_lumen_nM);

//! Rate laws

//!!V_abs_drug

V_abs_drug=k_abs_drug*drug_int;

//!!V_deg_drug

V_deg_drug=PLASMA*(k_deg_drug*fup_drug*Drug_pls);

//!!V_pls_to_prf_drug

V_pls_to_prf_drug=Q_prf_drug*(fup_drug*Drug_pls-Drug_prf);

//!!V_GFR_drug

V_GFR_drug=GFR*fup_drug*Drug_pls;

//!!V_excretion_drug

V_excretion_drug=PLASMA*(k_exc_drug*fup_drug*Drug_pls);

//!!V_reabsorption_drug

V_reabsorption_drug=LUMEN*(k_reab_drug*Drug_lum);

//!!V_urine_drug

V_urine_drug=Q_urine*Drug_lum;

//! Differential equations

//for drug_int

F[1]=(-V_abs_drug);

//for Drug_pls

F[2]=(V_abs_drug-V_deg_drug-V_pls_to_prf_drug-V_GFR_drug-V_excretion_drug+V_reabsorption_drug)/PLASMA;

//for Drug_prf

F[3]=(V_pls_to_prf_drug)/Vd_prf_drug;

//for Drug_lum

F[4]=(V_GFR_drug+V_excretion_drug-V_reabsorption_drug-V_urine_drug)/LUMEN;

//for drug_urine

F[5]=(V_urine_drug);

//! Data comments

///voulumes-L, dose-mg, time-hour

///variables-mg/L, drug_urine=mg

//! Compartments volumes

LUMEN=0.0171;//{L}

PLASMA=2.75;//{L}

//! Initial concentration

drug_int=F_drug*Dose_drug;//{mg}//

Drug_pls=0;//{mg/L}//

Drug_lum=0;//{mg/L}//

drug_urine=0;//{mg}//

Drug_prf=0;//{mg/L}//

//! Kinetic parameters

//!! General

GFR=7.2;//{L/hour}//=120 ml/min

Q_urine=0.055;//{L/hour}

//!! Dapagliflozin

///

//!!! PK

//!!!! Input

///

Mr_drug=408.9;//{g/mol}//

period_drug=24;//{hour}//input

Dose_drug=10;//{mg}//input

///

//!!!! Properties

///

F_drug=0.78;//{-}//

fup_drug=0.062;//{-}//

abs_lag_drug=4.178544e-01;

k_abs_drug=4.696729e-01;

k_deg_drug=8.851162e+01;

Vd_prf_drug=2.045249e+03;

Q_prf_drug=1.752201e+02;

k_reab_drug=2.836986e+00;

k_exc_drug=0;

///

//!!! PD

///

IC50_sglt1_drug=936.8333;//{nM}//

IC50_sglt2_drug=1.83333;//{nM}//

IC50_sglt3_drug=190000;//{nM}//

IC50_sglt4_drug=6050;//{nM}//

IC50_sglt5_drug=515;//{nM}//

IC50_sglt6_drug=1300;//{nM}//

///
